# Engineering of Ancestors as a Tool to Elucidate Structure, Mechanism, and Specificity of Extant Terpene Cyclase

**DOI:** 10.1021/jacs.0c10214

**Published:** 2021-01-26

**Authors:** Karen Schriever, Patricia Saenz-Mendez, Reshma Srilakshmi Rudraraju, Natalie M. Hendrikse, Elton P. Hudson, Antonino Biundo, Robert Schnell, Per-Olof Syrén

**Affiliations:** † School of Engineering Sciences in Chemistry, Biotechnology and Health, Science for Life Laboratory, KTH Royal Institute of Technology, 114 28 Stockholm, Sweden; ‡ School of Engineering Sciences in Chemistry, Biotechnology and Health, Department of Fibre and Polymer Technology, KTH Royal Institute of Technology, 114 28 Stockholm, Sweden; § Department of Medical Biochemistry and Biophysics, Karolinska Institutet, 17 165 Stockholm, Sweden; ∥ Swedish Orphan Biovitrum AB, 112 76 Stockholm, Sweden; ⊥ School of Engineering Sciences in Chemistry, Biotechnology and Health, Department of Protein Science, KTH Royal Institute of Technology, 114 28 Stockholm, Sweden; ∇ Wallenberg Wood Science Center, Teknikringen 56−58, 100 44 Stockholm, Sweden

## Abstract

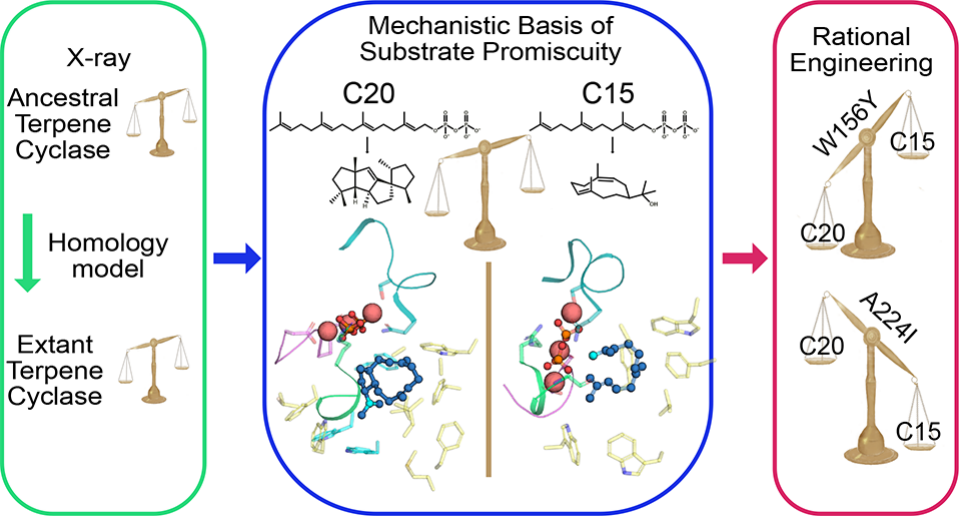

Structural information is crucial for understanding catalytic mechanisms and to guide enzyme engineering efforts of biocatalysts, such as terpene cyclases. However, low sequence similarity can impede homology modeling, and inherent protein instability presents challenges for structural studies. We hypothesized that X-ray crystallography of engineered thermostable ancestral enzymes can enable access to reliable homology models of extant biocatalysts. We have applied this concept in concert with molecular modeling and enzymatic assays to understand the structure activity relationship of spiroviolene synthase, a class I terpene cyclase, aiming to engineer its specificity. Engineering a surface patch in the reconstructed ancestor afforded a template structure for generation of a high-confidence homology model of the extant enzyme. On the basis of structural considerations, we designed and crystallized ancestral variants with single residue exchanges that exhibited tailored substrate specificity and preserved thermostability. We show how the two single amino acid alterations identified in the ancestral scaffold can be transferred to the extant enzyme, conferring a specificity switch that impacts the extant enzyme’s specificity for formation of the diterpene spiroviolene over formation of sesquiterpenes hedycaryol and farnesol by up to 25-fold. This study emphasizes the value of ancestral sequence reconstruction combined with enzyme engineering as a versatile tool in chemical biology.

## Introduction

Understanding how biosynthetic enzymes assemble chiral, complex products^1−3^ from simpler metabolites requires structural information on active site architectures. Spiroviolene synthase (SvS) from *Streptomyces violens* was first described by Rabe et al.^4^ and represents a bacterial class I diterpene cyclase that we were not able to produce in sufficient quantities for crystallization due to protein instability. A mechanism for spiroviolene formation has been suggested based on NMR experiments;^4^ yet due to the lack of a crystal structure it has remained unresolved how the enzyme chaperones the linear substrate in its active site during the cyclization reaction leading to the spirocyclic terpene. In this study, we have obtained a crystal structure of a stable and soluble reconstructed ancestor of SvS and used an engineered crystallized variant thereof as a template to derive a high-confidence homology model of extant SvS. Structural information enabled us to understand the molecular basis of substrate promiscuity and to engineer substrate specific variants of both ancestral and extant SvS.

A widely accepted hypothesis of enzyme evolution is that extant enzymes originate from biocatalysts that were adapted to different fitness landscapes,^5^ as was shown, e.g., for pancreatic artiodactyl ribonucleases that emerged from nondigestive ancestral ribonucleases.^6^ Amino acid or DNA sequences of putative ancestral enzymes can be computationally inferred using a phylogenetic tree of existing sequences and statistical models of evolution.^7,8^ Most experimentally characterized reconstructed ancestral enzymes have been associated with notably higher stability,^9−13^ and the reasons that may contribute to this observation are still being discussed.^9^ Since ancestral sequence reconstruction grants access to functional and robust enzymes without requiring structural input, it constitutes a powerful engineering approach^13,14^ alongside other existing methods to enhance stability and solubility.^15,16^ As protein crystallization benefits from high stability and solubility,^17,18^ it has been suggested that structures of ancestral enzymes could be used as a platform to approach the structures of extant enzymes that are challenging to study.^13^ Crystal structures of reconstructed enzymes have been presented and been used together with biochemical analyses to derive knowledge about principles of molecular evolution.^11,19−25^ We anticipated that a reconstructed thermostable ancestral SvS (SvS-A2)^26^ would be more amenable to crystallization and could serve as structural template to derive a homology model of the extant enzyme. In this study, we show that further engineering of a surface patch in the reconstructed ancestor was necessary to achieve this goal.

Terpene cyclases such as SvS form a variety of complex multicyclic compounds with potent biological activities via carbocationic, electrophilic cyclization cascades of relatively simple substrates, either by metal-ion assisted release of an allylic pyrophosphate (class I mechanism) or by protonation of an oxirane/ene-functionality (class II mechanism).^27^ Different terpene cyclases act on the same set of linear substrates which are classified according to their isoprene-unit content (C5, hemi-; C10, mono-; C15, sesqui-; C20, di-; C25, sester-; C30, tri-; C40, tetraterpenes).^28^ Except for a few strictly conserved catalytic motifs, terpene cyclases that share a similar active site architecture have been shown to exhibit low sequence identity.^27^ In addition, it has been demonstrated that categorizing plant sesquiterpene cyclases by sequence did not correlate with unique chemical fingerprints of the afforded products.^29,30^ Generating homology models of structurally unresolved terpene cyclases, based exclusively on sequence identity to other crystallized enzymes, may thus constitute a challenge. For the case of SvS this is exemplified by the fact that building a homology model based on the enzyme with closest sequence identity in the Protein Data Bank—bacterial sesquiterpene cyclase selinadiene synthase (*SdS*)^31^—resulted in lower model confidence for cofactor binding regions (discussed below). In contrast, we hypothesized that crystal structures of inherently related putative ancestral terpene cyclases could be more easily accessible and present reliable templates for homology modeling of their extant counterparts. We have applied and critically evaluated this concept using X-ray crystallography in conjunction with enzyme engineering, homology and molecular modeling, docking studies, and in vitro enzymatic assays. In this way, we obtained the structures of SvS-A2 and an engineered surface variant thereof. The latter proved to be a superior template for construction of a high-confidence homology model of extant SvS. Using the obtained crystal structures and derived homology model we could shed light on the structure–activity relationship of the class I cyclization cascade displayed by SvS. By studying both farnesyl pyrophosphate (FPP, C15) and geranylgeranyl pyrophosphate (GGPP, C20) cyclization trajectories, we could identify residues involved in modulating substrate promiscuity in SvS.

Due to their diverse and appealing properties such as fragrances or antiviral, antimicrobial, and anticancer activity, terpenes have gained increasing industrial attention in the past decades.^27,28^ Moreover, they constitute an underexplored renewable carbon source amenable for generation of biofuels, biochemicals, and polymers.^32^ Besides different possible initial cyclization trajectories,^33^ which are guided by substrate prefolding, terpene diversity is introduced by ring expansions and methyl- or hydride shifts that accompany the propagation of the carbocation upon ring closure.^27,28^ This versatility in stereospecific C–C bond formation is a sought-after property in synthetic chemistry and expansion of accessible chemical terpene space has been studied.^34^ As chemical synthesis of polycyclic synthons can be challenging, efforts in developing processes for enzymatic or microbial production of terpenes and terpene-derived products attract significant interest.^35−37^ In this context several enzyme engineering methods have been applied to study and optimize terpene cyclases, such as domain-swapping studies, directed evolution approaches, and rational redesign targeting a combination of residues conveying functional plasticity.^38−41^

Terpene cyclases are active in secondary metabolism and have *k*_cat_ values that are often in the range of or less than one turnover per minute.^41,42^ Engineering specificity in terpene cyclases is challenging and was shown to benefit from structural information to guide enzyme redesign,^40,41^ as small perturbations in the active site architecture can affect substrate prefolding and generation of discrete, transient carbocationic species, impacting both substrate^42^ and product specificity.^43−45^ We aimed at using the generated structural information as basis for controlling substrate specificity in SvS in a targeted manner. We designed specific ancestral variants that retained thermostability and solved the crystal structures of representative variants. Moreover, the identified specificity switches could be functionally transferred to the extant enzyme, highlighting the utility of the ancestral scaffold as model for improvement of a cognate extant enzyme. These results demonstrate the suggested utility of reconstructed ancestral enzymes as scaffolds for further engineering in synthetic biology applications.^13^ In summary, this study highlights how reconstruction of an ancestral biocatalyst and the derived structural information in concert with enzyme engineering broadens the structure activity relationship-based comprehension of extant biosynthetic enzymes.

## Results and Discussion

### Crystal Structure of Ancestral Terpene Cyclase

We were unable to crystallize extant SvS (*SvS-WT*) from *S. violens* due to low expression yields in *Escherichia coli* BL21(DE3) and the enzyme’s susceptibility to aggregate, when being concentrated to the levels required for crystallographic studies. We previously reported a hypothetical ancestor of SvS—*SvS-A2* (Figure S1)^26^—which shares 77% sequence identity with the extant enzyme.

This ancestor showed both enhanced solubility and elevated thermal stability over SvS-WT (Figure S2), which is why we reasoned that SvS-A2 would be more amenable to structural studies and represent a suitable structural template for homology modeling of SvS-WT.

**Figure 1:**
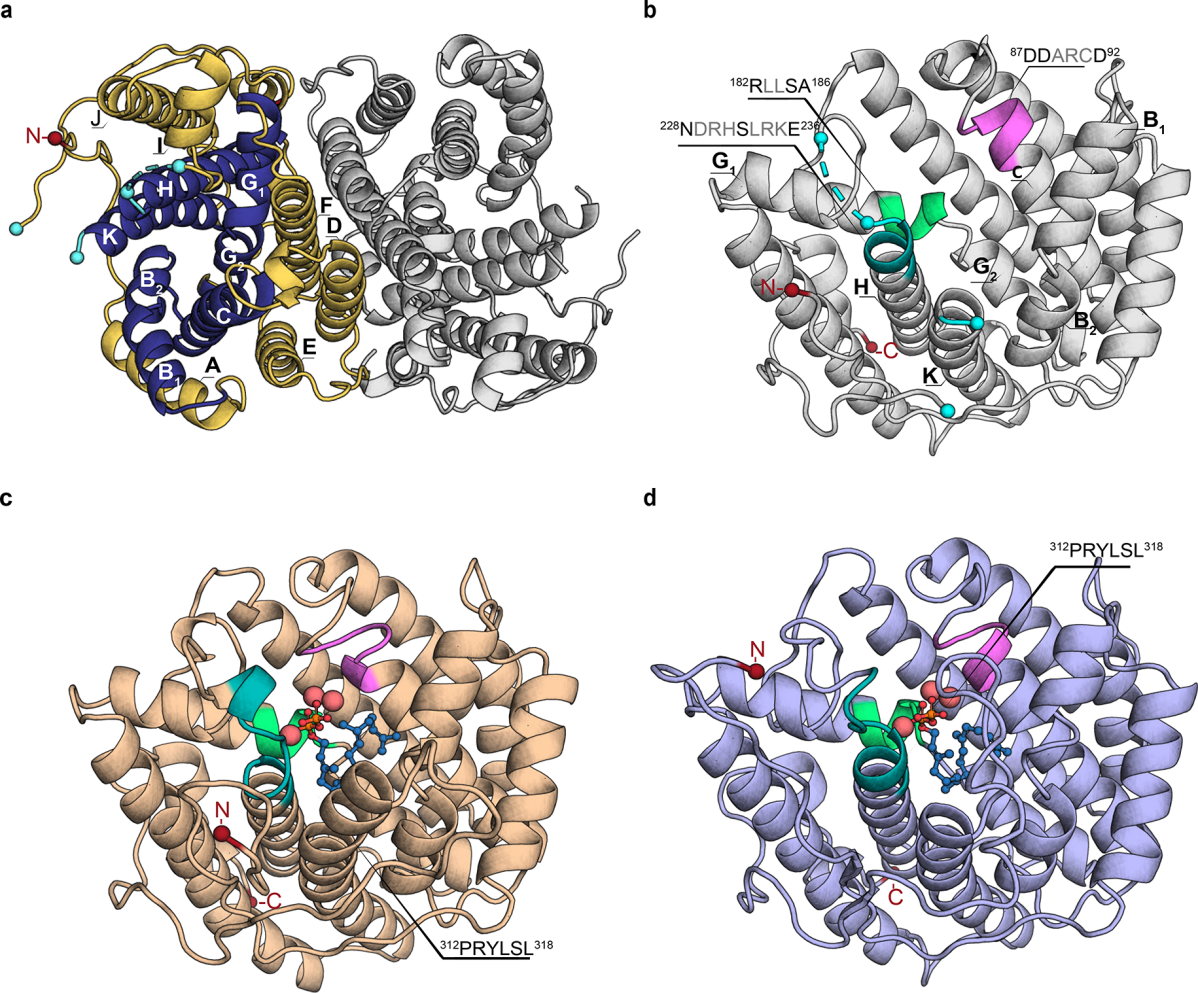
Crystal structure of SvS-A2 and homology model of SvS-WT. (a) Top view of full dimeric crystal structure of reconstructed ancestral spiroviolene synthase SvS-A2, PDB-ID: 6TBD (2.30 Å). In one monomer, helices B, C, G, H, and K forming the active site cavity are shown in dark blue, peripheral helices A, D, E, F, I, and J are shown in gold. The first resolved N-terminal residue (Asp09) is shown as a red sphere. Residues that are flanking unresolved loops (Leu233/His241 and Arg311/Pro329) are shown as cyan spheres. (b) Front view of a monomer of the SvS-A2 crystal structure. The DDxx(x)D motif is shown in violet, NSE motif in teal, effector motif in light green. (c) Final model of SvS-A2 with missing loops and the trimetal ion cluster (shown as enlarged pink spheres) modeled and GGPP substrate (shown as blue sticks and spheres) docked as described in the Supporting Methods section. (d) Substrate-docked homology model of extant SvS-WT (monomeric) based on a surface variant of SvS-A2 (sequence-identity 78%). Motifs and additional molecules are shown as described in (c). The modeled C-terminal end of helix K (^312^PRYLSL^318^) shows considerable structural changes between SvS-A2 and SvS-WT.

In a first step, the unliganded, metal-free crystal structure of SvS-A2 was determined to 2.30 Å resolution (PDB-ID: 6TBD, Figure 1, Tables 1 and S1). SvS-A2 forms a homodimer in solution, which was confirmed by size exclusion chromatography (Figure S2b), consistent with the dimer found in the asymmetric unit of the crystal unit cell (Figure 1a). Each monomer harbors one active site which points away from the dimer interface, resulting in an overall antiparallel arrangement. Likewise, SvS-WT was found to be dimeric in solution (Figure S2b), excluding a major impact of the ancestral mutations on dimerization.

**Table 1:** X-ray Diffraction Data Statistics and Model Parameters

protein variant	SvS-A2	SvS-A2	SvS-A2 (205–209)	SvS-A2	SvS-A2
		(W79F, G83L)	(DREMH/AQDLE)	(A224I)	(W156Y)
PDB code	6TBD	6TJA	6TIV	6THU	6TJZ
beamline	ESRF/ID23-1	MAX-IV/Biomax	MAX-IV/Biomax	MAX-IV/Biomax	MAX-IV/Biomax
space group	*P*2_1_2_1_2_1_	*P*2_1_2_1_2_1_	*P*2_1_2_1_2_1_	*P*2_1_2_1_2_1_	*P*2_1_2_1_2_1_
Unit cell^a^					
*a*, *b*, *c* (Å)	75.3, 105.5, 105.5	74.8, 104.9, 108.7	74.1, 104.2, 108.2	74.9, 105.1, 108.3	74.9, 104.6, 109.2
a, β, γ (deg)	90.0, 90.0, 90.0	90.0, 90.0, 90.0	90.0, 90.0, 90.0	90.0, 90.0, 90.0	90.0, 90.0, 90.0
resolution (Å)	42.20–2.30	29.41–2.27	29.66–2.38	43.02–2.6	40.66–2.4
	(2.38–2.30)	(2.34–2.27)	(2.48–2.38)	(2.72–2.6)	(2.49–2.40)
no. of unique reflections	37596 (3635)	40139 (3637)	34458 (4109)	26961 (3237)	34301 (3555)
*I*/σ(*I*)	9.0 (2.9)	9.7 (2.7)	7.7 (3.0)	15.1 (2.4)	10.8 (1.9)
redundancy	5.2 (5.2)	5.6 (5.6)	6.7(6.7)	6.6 (6.9)	5.6 (5.0)
completeness (%)	99.1 (99.3)	99.8 (99.6)	99.8 (98.8)	99.9 (100.0)	100.0 (99.9)
*R*_merge_	0.139 (0.677)	0.100 (0.528)	0.129 (0.526)	0.072 (0.700)	0.064 (0.642)
*R*_pim_	0.075 (0.406)	0.050 (0.264)	0.057 (0.234)	0.033 (0.312)	0.031 (0.353)
CC(1/2)	0.991 (0.785)	0.976 (0.853)	0.994 (0.855)	0.999 (0.801)	0.999 (0.739)
Wilson *B*-value (Å^2^)	40.3	30.0	26.0	50.7	61.5
Refinement					
*R*	0.184	0.185	0.187	0.19	0.180
*R*_free_	0.218	0.220	0.21	0.216	0.221
Number of atoms/*B*-factor Å^2^					
overall	5381/36.0	5360/41.1	5545/32.2	5206/63.0	5265/65.9
protein	5107/36.1	5121/42.0	5159/31.8	5161/66.8	5206/69.2
PEG	n/a	21/40.9	94/45.5	n/a	n/a
water	274/34.0	218/35.7	292/32.4	45/55.5	59/59.4
rmsd from ideal geometry					
Bond length (Å)	0.0160	0.0098	0.0107	0.0081	0.0075
Bond angles (deg.)	1.58	1.63	1.70	1.57	1.53
Ramachandran Plot *N* of residues (%)					
in preferred regions	619 (99.20%)	623 (98.26%)	622 (97.19%)	607 (95.44%)	627(97.21%)
in allowed regions	5 (0.80%)	11 (1.74%)	17 (2.66%)	29 (4.56%)	18 (2.79%)
outliers	0 (0.0%)	0 (0.0%)	1 (0.16%)	0 (0.0%)	0 (0.0%)

a Values in parentheses are for the highest resolution shell.

The monomer represents the typical isoprenoid synthase α-fold (InterPro entry IPR008949), which is comprised of 11 antiparallel α-helices with the active site embedded between helices B, C, G, H, and K that form an inner circular arrangement (shown in dark blue in Figure 1a). The signature metal-binding motifs of terpene cyclases are located on helix C (aspartate-rich motif ^87^**DD**ARC**D**^92^, shown in violet cartoon in Figure 1) and on helix H (NSE motif ^228^**N**DRH**S**LRK**E**^236^, shown in teal cartoon in Figure 1).^27^ Electron densities in the termini, two loops that were unresolved (7 and 17 residues, respectively), and the canonical trimetal ion cluster were modeled as described in the Supporting Methods, and the substrate GGPP was computationally docked into the active site of one monomer (Figure 1c). One of the two modeled loops comprises a conserved RY-dimer of bacterial terpene cyclases in the C-terminal end of helix K (^312^PRYLSL^318^), which adopts a helical structure in the GGPP-docked model (Figure 1c).^46^ The equivalent segment is unresolved in most crystallized apo-terpene cyclases and closes the active site in substrate (or analogue)-bound crystal structures and has been suggested to be involved in substrate binding.^31,47^

The terpene cyclase of resolved structure with highest sequence identity to SvS-A2 is the sesquiterpene cyclase selinadiene synthase,^31^ which shares 31.2% and 30.1% sequence identity with SvS-A2 and SvS-WT, respectively. In SdS, a catalytic effector motif consisting of a phosphate sensor, a linker-residue, and an effector residue was described to mediate an induced-fit conformational rearrangement upon substrate binding,^31^ positioning the backbone carbonyl of the effector residue in closer proximity to the substrate. A functionally equivalent motif (Arg182, Ser185, and Ala186, shown in light green in Figure 1) is present in SvS-A2. Since this motif exhibits a similar orientation in the unliganded crystal structure as in the substrate-docked model (local Cα root-mean-square deviation (rmsd) of 0.22 Å over five residues) as well as substrate-bound SdS (Cα rmsd 0.30 Å over five residues), an equivalent induced fit rearrangement would likely occur to a lesser extent in SvS-A2.

The fact that the ancestral enzyme yields a crystal structure is in line with the presumption that ancestral enzymes could be more amenable for structural characterization,^13^ and in agreement with reports of other metabolic enzymes for which ancestral crystal structures have been reported, such as a bacterial pyruvate decarboxylase and mammalian cytochrome P450.^20,48^

### Using Ancestral Enzyme Structure as Template for Homology Modeling of Extant SvS

Terpene cyclases that share a similar active site fold have been shown to exhibit low sequence identity.^27^ Generating homology models of structurally unresolved terpene cyclases that appropriately reflect substrate binding based exclusively on sequence identity to other crystallized enzymes can thus constitute a challenge.

In a recent study reporting structures of reconstructed membrane-bound mammalian flavin monooxygenases, for which no extant structures are resolved, it was suggested that X-ray structures of ancestral enzymes can be considered model structures for extant enzymes.^19^ In line with this concept, we sought to build a homology model of SvS-WT based on SvS-A2 as a template, supported by their sequence identity of 77%, which is well above the limit of 30% that allows for prediction with an accuracy comparable to a low to medium resolution crystal structure.^49^ To this end, we used the substrate-docked model of SvS-A2 (Figure 1c) as single template structure in the homology modeling process.

The model was primarily evaluated by the *Z*-score, which reflects the amount of standard deviations with which the calculated normalized energy of the model deviates from that of an average high-resolution X-ray structure (a negative score indicating that the homology model is considered nonoptimal in the corresponding region). Unexpectedly, the obtained homology model (SvS-WT-Hom1) displayed a suboptimal average *Z*-score for dihedral angles (*Z*-score of −0.429, Table S2). Moreover, the local *Z*-score was not optimal for regions that are involved in metal binding (*Z*-score of up to −2.0 in the DDxx(x)D motif and −1.5 in the NSE motif, Figure S3a). Complementary evaluation of protein geometry and molecular contacts by Verify 3D analysis highlighted that only 87.0% of residues scored over the quality threshold of ≥0.2 in the 3D-1D profile.

**Figure 2:**
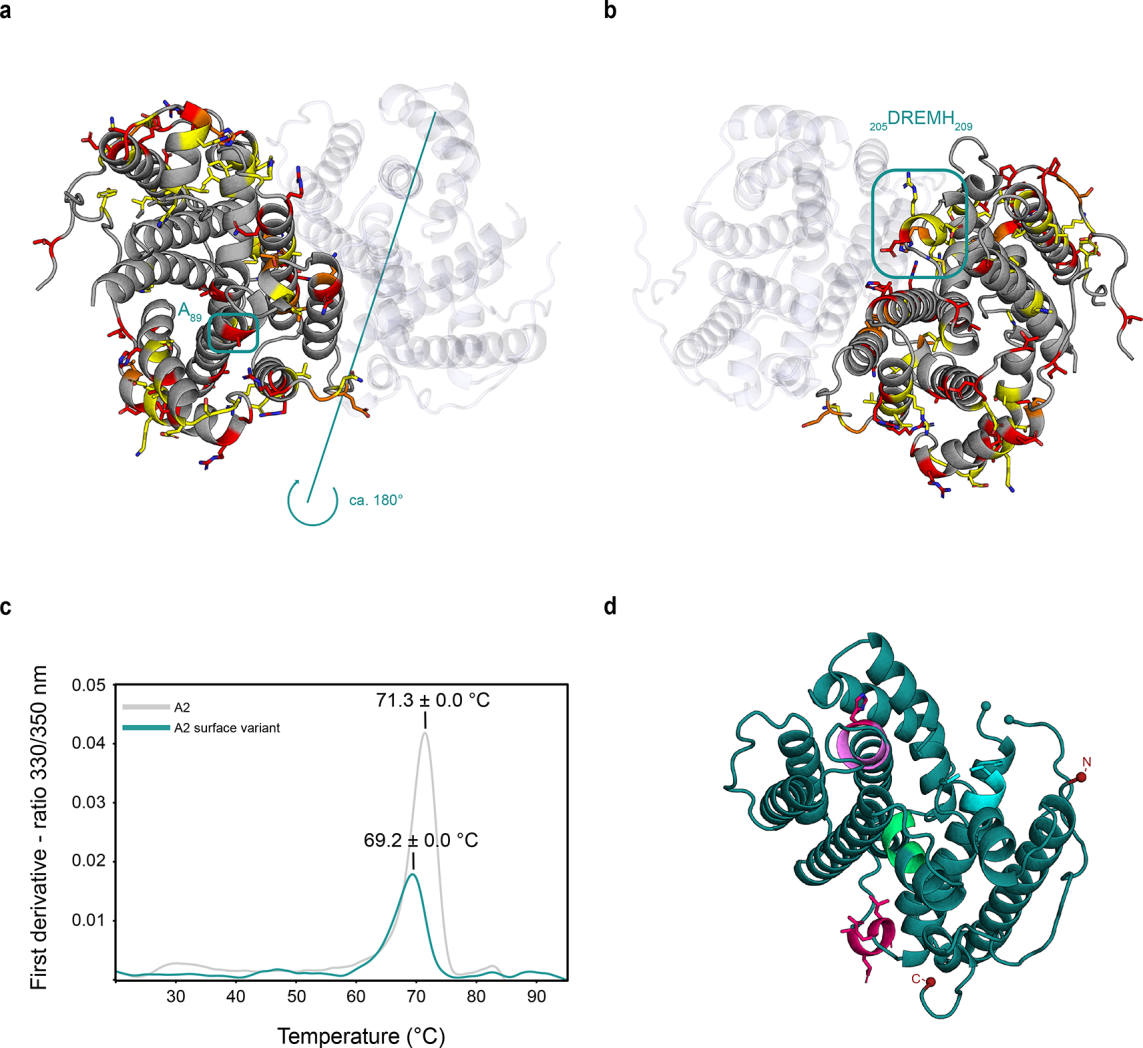
Distribution of ancestral mutations on SvS-A2 surface. (a) The backbone of the SvS-A2 crystal structure is shown in gray. Ancestral mutations (i.e., positions that differ between SvS-A2 and SvS-WT) are shown as sticks; yellow, exchanges with residues that have similar properties (e.g., Asp/Glu, Lys/Arg); orange, exchanges with residues that have less similar properties (e.g., Glu/His, Ser/Asp); red, exchanges with residues with nonsimilar properties (e.g., Pro/Ala, Thr/Arg). The position of Ala89 is enclosed by a blue box and labeled. (b) Structure in (a) rotated by approximately 180°. The position of the consecutive residues of the surface patch (205DREMH209) is enclosed by a box and labeled. (c) Thermal melting curves of the reconstructed ancestral enzyme and surface variant thereof as determined by nanoDSF (*T*_m_ SvS-A2 71.3 ± 0.03 °C, *T*_m_ SvS-A2 surface variant 69.2 ± 0.01 °C). (d) Crystal structure of SvS-A2 surface variant (205–209:DREMH/AQDLE_Ala89His). Colors of motifs as indicated in Figure 1, mutated positions are shown as pink sticks. The protein crystallizes as a dimer, but only one monomer is shown for better visibility.

We therefore realized the need to optimize the ancestral structure used as input for homology modeling by enzyme engineering. The distribution of ancestral mutations across the protein fold was inspected with the aim to identify individual clusters of ancestral mutations that may be exchanged back to wild-type residues to further increase sequence identity without compromising protein stability. While the active site lining is virtually conserved between SvS-A2 and SvS-WT (Table S3), the majority of ancestral mutations are hydrophilic and dispersed on the surface of SvS-A2 (Figure 2a,b). In particular, we noticed a surface patch of five consecutive ancestral mutations (^205^DREMH^209^) in the turn directly preceding helix H (Figure 2b). This patch is located 17.9 Å away from the carbonyl atom of the effector residue (Ala186), so that it unlikely impacts the activity of SvS-A2. Moreover position 89 in the catalytic DDxx(x)D motif occupies a His in SvS-WT, but an Ala on the surface of SvS-A2. It has previously been described that individual surface mutations can impact the crystallization of terpene cyclases by reducing surface entropy.^50^ Exchanging these six residues on the surface back to the corresponding wild-type residues (205–209:DREMH/AQDLE and Ala89His) resulted in a surface variant of SvS-A2 with similar stability (Figure 2c), despite an overall reduced expression yield. A dimeric crystal structure of this surface variant could be determined at 2.38 Å resolution (PDB-ID: 6TIV, Table 1, Figure 2d) with a Cα rmsd of 0.37 Å over 624 atoms to the parental SvS-A2 crystal structure.

Moreover, five additional residues were resolved in the 17-residue segment of the capping loop that contains ^312^PRYLSL^318^ as shown in Figure 1. Therefore, we anticipated that the surface variant may constitute a more suitable structural template for homology modeling of SvS-WT, especially with respect to adequately capturing substrate binding.

Using the surface variant as a template (monomeric with modeled loops, metal cluster and GGPP) resulted in a homology model (SvS-WT-Hom2) with overall higher sequence identity as well as optimal average scores for dihedral angles (*Z*-score of +0.541, Table S2). Specifically, the local *Z*-score slightly improved for the catalytic DDxx(x)D and NSE motifs (Figure S3b, arrows), even though the local *Z*-score for the RY-dimer decreased (Figure S3b). A Ramachandran plot of SvS-WT-Hom2 showed that 97.8% of the residues are located in the most favored region, while only 1.9% and 0.3% are placed in the allowed and disallowed regions, respectively. The quality of SvS-WT-Hom2 was further confirmed by Verify 3D analysis with 95.0% of residues scoring over the quality threshold of ≥0.2 in the 3D-1D profile and an ERRAT score of 98.9%.

For comparison, a homology model was also constructed using selinadiene synthase as a template with the same modeling parameters. The resulting homology model (SvS-WT-Hom3) contains several gaps, some of which are located in the loop containing the RY-dimer and yields a negative *Z*-score of −0.266 for dihedral angles (that excludes 15 terminal residues,Table S2). Structural differences to the other models are mostly located in the upper part of the active site (Figure S3c) and are associated with lower local *Z*-scores.

The generated homology models differ in the conformation of the modeled capping loop K. In SvS-WT-Hom1 (as well as in SvS-A2) this sequence adopts a helical fold and points to the surface of the enzyme, appearing to prevent full closure of the active site cleft (Figure S3a, top row). In contrast, this modeled segment in SvS-WT-Hom2 corresponds to an unstructured loop that folds back onto the active site (Figure S3b, top row). This difference observed in the protein backbone is guided by the five additional residues that were resolved in the crystal structure of the surface variant.

Overall, we considered the homology model derived from the SvS-A2 surface variant (SvS-WT-Hom2) more suitable for mechanistic analysis of the extant enzyme. SvS-WT-Hom2 was finally energy minimized using the AMBER force field (Figure 1d) and used in subsequent structural comparisons between SvS-A2 and SvS-WT. In summary, the structural differences observed between SvS-A2 and SvS-WT-Hom2 suggest that the ancestral enzyme structure itself cannot be considered a direct model for SvS-WT per se. Moreover, the fact that confidence in the model improves when using the surface-variant as template highlights that it is beneficial to optimize the extent of sequence identity when using ancestral enzymes as structural models.

### Promiscuous Sesqui-/Diterpene Cyclase Activity in Ancestral and Extant SvS

**Figure 3:**
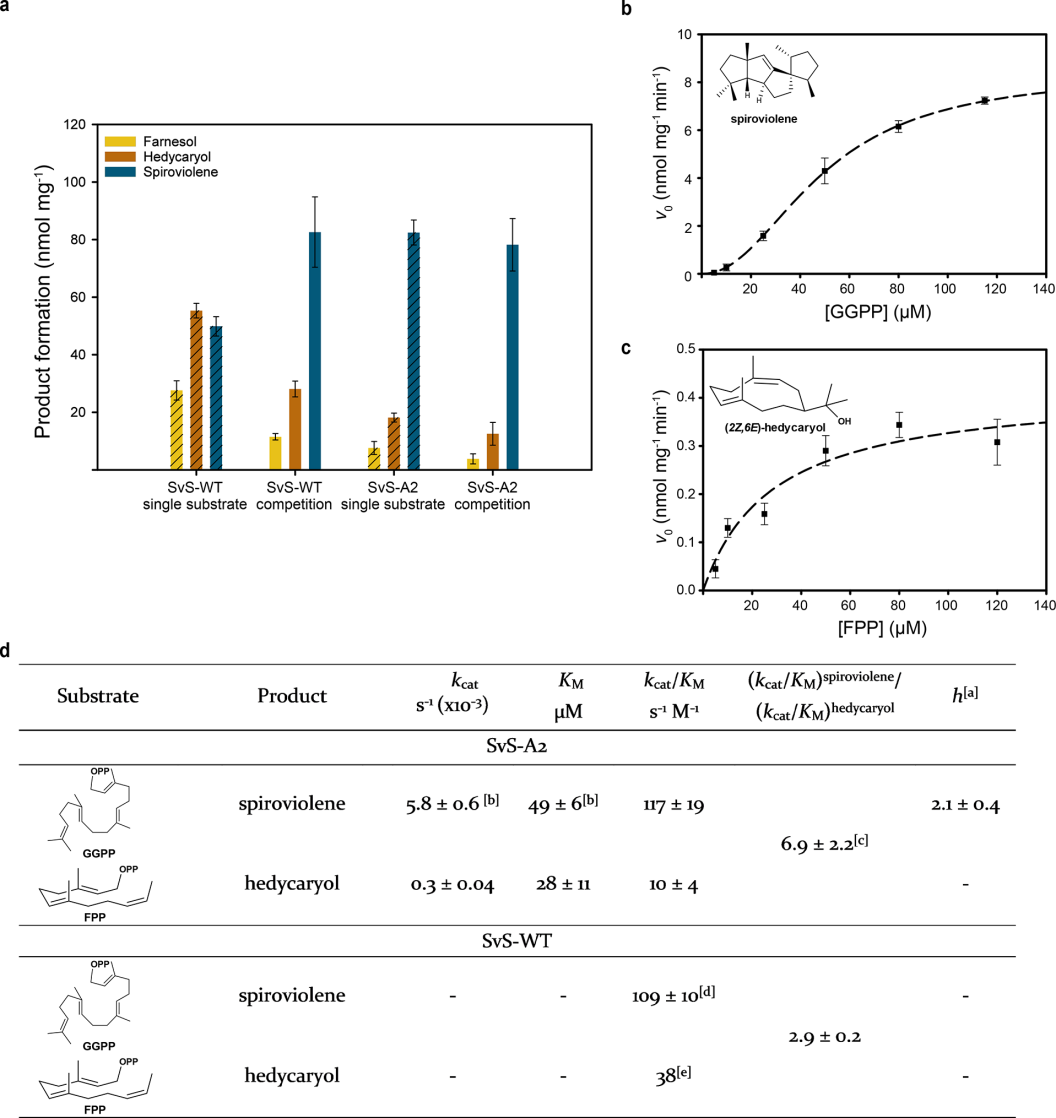
Kinetic analysis of SvS-A2 and SvS-WT. (a) In vitro product formation by ancestral and extant SvS (2 μM) using single substrate (60 μM of FPP or GGPP, striped bars) or an equimolar mix of both (60 μM, each, filled bars). The reactions were incubated at 30 °C for 3 hours. Specific production of farnesol is shown as yellow, hedycaryol as orange, and spiroviolene as dark blue bars. Initial rates for SvS-A2 are plotted against the substrate concentration for formation of spiroviolene (b) and hedycaryol (c) using 0.5 μM enzyme. Cooperative and standard Michaelis–Menten curve fits shown as dashed lines (*R*^2^ values of 0.977 and 0.792, respectively). Error bars represent the standard error of triplicates. (d) Kinetic parameters derived from panel (a) and the equations fitted to the data in (b,c). Differences may occur due to rounding. ^*a*^Cooperativity described by the Hill coefficient *h* (eq 1, Supporting information). ^*b*^The *k*_cat_ value for SvS-A2 catalyzed spiroviolene formation is close to the value we previously reported (6.5 ± 0.6 × 10^–3^ s^–1^).^26^ The *K*_M_ value for GGPP reported herein is twice as high as previously reported (27 ± 0.6 μM),^26^ likely due to the use of different reaction conditions. The higher *K*_M_ observed herein explains why no cooperative behavior was previously observed. ^*c*^The value is obtained from competition experiments and is rather close to the predicted value of 11.7, which is calculated from the individual (*k*_cat_/*K*_M_) values. ^*d*^Taken from Hendrikse et al.^26^ for comparison. ^*e*^Value inferred from previously reported *k*_cat_/*K*_M_ values and observed relative second order rate constants in the competition experiment.^26^

Ultimately, the motivation for using reconstructed ancestral enzymes to generate structural models of extant biocatalysts lies in the expectation that ancestors retain the extant enzymes’ reaction mechanisms. We therefore evaluated enzymatic activity and specificity of both ancestral and extant SvS alongside one another and found that both enzymes accepted FPP and GGPP as substrates (Figure 3).

The major sesquiterpene product was identified as elemol by gas chromatography mass spectrometry (GC-MS) analysis, based on spectral comparison (95% identity to NIST-library) and linear retention index analysis (Figures S4a–c and S4i).^51,52^ In line with several studies that described elemol as thermal rearrangement product of hedycaryol,^52−54^ the peak for elemol almost completely disappeared when reducing the injection port temperature (Figure S5a,b) and a new broad peak with the top *m*/*z* values of hedycaryol arose next to the elemol-peak; a rearrangement that has been previously used in confirming hedycaryol (Figure S5c).^54^ Farnesol was further identified as one of the sesquiterpene side products based on analysis of a reference standard (Figures S4d,e and S4i). The major diterpene product was assigned to be spiroviolene by mass spectrometry analysis and comparison to previously published data (Figures S4f–h and S4i).^4^

Strict diterpene substrate specificity has been reported for SvS-WT by Rabe et al.^4^ and us.^26^ However, upon incubation with 60 μM FPP using phosphate-free buffer (50 mM tris(hydroxymethyl)aminomethane-HCl, pH 7.4), 2 μM of freshly purified and desalted enzyme at 30 °C, it was observed that the extant enzyme in fact showed higher activity with FPP than GGPP (striped bars, Figure 3a, left). This deviation is likely due to different reaction conditions: in vitro assays of the wild type were previously performed using sodium phosphate buffer and higher concentrations of FPP.^26^ When using 60 μM of both substrates in a competition setup, SvS-WT was more active with GGPP but showed promiscuous sesqui-/diterpene activity (Figure 3a, nonstriped bars), generating larger quantities of sesquiterpene products (hedycaryol and farnesol) than SvS-A2 (Figure 3a, nonstriped bars).

In order to evaluate whether the generated structural homology model of SvS-WT accurately reflects these substrate preferences, binding energies between the holoenzymes (SvS-A2 and SvS-WT-Hom2, including the metal ion cluster) and both substrates were calculated (Table S4). The binding preference for GGPP over FPP was found to be slightly less pronounced in SvS-WT-Hom2 (Δ_GGPP-FPP_Δ*G*_bind_ −8.72 kcal mol^–1^) than in SvS-A2 (Δ_GGPP-FPP_Δ*G*_bind_ −10.69 kcal mol^–1^), in qualitative agreement with the experimental results.

Studying enzyme kinetics with individual substrates revealed that SvS-A2 exhibits well-defined cooperativity for spiroviolene production (Figure 3b) with a Hill-coefficient of 2.1 ± 0.4, whereas it was previously shown that SvS-WT could not be saturated with GGPP.^26^ The observed cooperativity may potentially reflect an induced fit mechanism within the dimeric ancestral protein, which is absent in extant SvS due to the accumulated surface mutations. SvS-A2 further exhibits classic Michaelis–Menten kinetics for the formation of hedycaryol (Figure 3c) from FPP. The values for kinetic parameters observed in SvS are within the range of other class I terpene cyclases^41,42^ and may reflect product release being the rate-limiting step, as has been determined for trichodiene synthase by pre-steady-state kinetics.^55^

Taken together the comparisons show that both SvS-WT and its reconstructed ancestor SvS-A2 act on the same substrates and show minor differences in specificity, which are captured in the generated homology model. Our data does not rule out that different steps may be rate-limiting between SvS-A2 and SvS-WT and the observed differences in their kinetic behavior highlight a possible limitation of the approach outlined herein.

### Mechanistic Insights into Promiscuous Sesqui-/Diterpene Activity

The reaction mechanism for spiroviolene formation has been suggested based on NMR experiments.^4^ Yet, due to the absence of an enzyme crystal structure, it has remained elusive which residues in the SvS active site are responsible for directing the suggested ring formations. We aimed to assess whether the obtained structural information would allow to pinpoint individual active site residues involved in carbocation stabilization in extant and ancestral SvS in order to understand the molecular basis of substrate promiscuity.

To this end molecular modeling was used following the initially suggested reaction mechanisms for spiroviolene formation as well as that of hedycaryol synthase (HecS) from *Kitasatospora setae* (35.5% sequence similarity to SvS-A2).^4,51^

**Figure 4:**
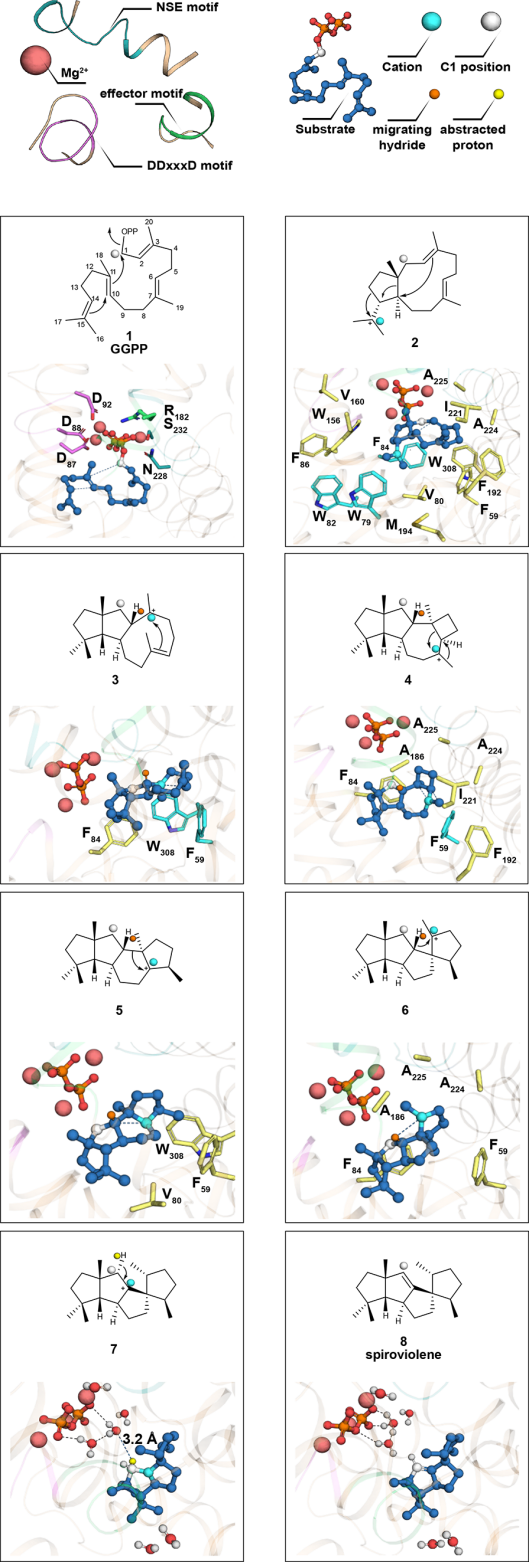
Snapshots of the SvS-A2 catalyzed cyclization of GGPP to spiroviolene. The same interactions are valid for the SvS-WT homology model but are not shown for clarity. Key distances for bond forming/breaking reactions are indicated as blue dotted lines and respective distances for the reconstructed ancestral and the extant enzyme are given in Table S5. The proposed electron flow is represented with conventional arrows in the 2D-depiction. Residues involved in complexing the metals and pyrophosphate in **1** are shown as sticks but are omitted for clarity in other panels. The full hydrophobic cage around the intermediate is shown as sticks in **2** and for clarity only residues in close proximity of the cation are shown as sticks in the following panels. Individual residues involved in π-interactions are highlighted as cyan sticks and the hydrogen that is transferred in the penultimate reaction step is shown as orange sphere. For deprotonation of intermediate **7** water molecules within a radius of 9 Å of the ligand are shown; the abstracted hydrogen atom (distance of 3.2 Å) is shown as yellow sphere.

Starting from the product-docked model of SvS-A2 and SvS-WT-Hom2, bonds were manually broken and formed to obtain the different intermediary cations and finally the substrate GGPP in the correct prefolded conformations, as described in the Supporting Methods section. The snapshots of the different intermediates interacting with the SvS-A2 active site are shown in Figure 4. Differences in key distances for bond forming and breaking mechanistic steps between ancestral and extant SvS are summarized in Table S5. After pyrophosphate release, intermediate **2** is stabilized by several hydrophobic residues lining the active site. In particular, clear cation-π interactions are observed with Trp79 and Phe84, which are located on helix C. The cation is also stabilized by the backbone carbonyl of Gly83 and may undergo additional π interactions with Trp82 and Trp156 (Figure 4). In contrast, intermediate **3** is stabilized by π interactions with Phe59 and Trp308, which are located on the opposite wall of the active site on helices B and K. Subsequent carbocationic intermediates are mostly stabilized by Phe59 and the backbone carbonyl of Ala186 (the effector residue).

Despite the fact that all intermediates can plausibly be accommodated in the obtained structures, we noticed subtle differences to the suggested mechanism,^4^ including the configuration of the C2-atom in intermediate **3**. According to our model, the C2-atom resides in *R* configuration, with the hydrogen (highlighted orange in Figure 4) pointing *syn* to the methyl group at C11. This hydride is transferred in the penultimate reaction step (intermediate **6**), which is facilitated by its orientation. Moreover, our model suggests that the final deprotonation at C1 in **7** to form spiroviolene (**8**) cannot be directly mediated by the pyrophosphate anion, as previously suggested,^4^ because the distance between the closest negatively charged oxygen and the proton is 5.7 Å in SvS-A2 (4.0 Å in SvS-WT-Hom2). Instead, it was observed that the suggested *pro*-R deprotonation is facilitated by suitable positioning of a water molecule (CH···O_wat_ distance of 3.2 and 2.5 Å in SvS-A2 and SvS-WT-Hom2, respectively). Due to the presence of additional water molecules within a radius of 9 Å from the ligand a Grotthuss mechanism^56^ for facilitated proton transport is likely.

Recently, an alternative configuration of the product spiroviolene has been suggested, in which the methyl-group at C3 points *syn* to the methyl-group at C19.^57^ On the basis of our structural data, a *syn* configuration of the methyl-groups would also be plausibly accommodated in the active site structure (**7′** in Figure S6). Visual inspection shows that formation of **7′** from **6** would be feasible. Alternative mechanistic steps for the generation of **7′** from **3** were also very recently proposed.^58^

In the same manner, snapshots of the FPP conversion to (2*Z*,6*E*)-hedycaryol in SvS-A2 and SvS-WT were generated. The snapshots for SvS-A2 are shown in Figure S7 with corresponding key distances in SvS-A2 and SvS-WT given in Table S6. The reaction mechanism for hedycaryol formation requires charge stabilization in a more confined area of the active site pocket, which is mediated by interactions with the pyrophosphate moiety and residues on helix C (Phe84, which is conserved in SvS-A2, HecS, and SdS). In SvS-WT the initial farnesyl cation (**10**) is pushed closer toward helices G and H than in SvS-A2, resulting in loss of hydrophobic contacts with aromatic residues on helices B2 and K, which is in line with substrate binding affinity calculations. On the basis of the observed distances, the backbone carbonyl of Ser185 may stabilize cation **10** in SvS-WT (Ala186 in SvS-A2).

Taken together, our results can explain how both extant and reconstructed ancestral SvS can act as dual functioning sesqui-/diterpene cyclases. The described diterpene reaction mechanism^4,58^ involves major charge rearrangements (Figure 4), which requires aromatic residues to sequentially stabilize the intermediate cations via π-interactions on both walls of the binding pocket (such as Trp79, Trp82, Phe84, and Trp156 on helices C/F and Phe59 and Trp308 on helices B/K), whereas the sesquiterpene reaction mechanism occurs in a more focused region of the active site. Interestingly, the tryptophan corresponding to Trp308 that we propose to be involved in the diterpene formation, is conserved in SdS and HecS, which are both sesquiterpene cyclases, even though a direct involvement of this tryptophan in the sesquiterpene reaction mechanism has been excluded.^31,51^ Positions corresponding to Trp79 and Trp156 on the other wall of the active site in SvS-A2 are involved in diterpene cation stabilization and are also occupied by aromatic residues in the sesquiterpene cyclases HecS and SdS (Table S3). These observations raise the question whether these related enzymes may also be able to act as promiscuous sesqui-/diterpene cyclases with individual residues directing substrate specificity.

### Structure-Guided Engineering of Specificity in Ancestral and Extant SvS

Several studies have capitalized on ancestral sequence reconstruction to engineer stable and promiscuous biocatalysts.^10,59,60^ On the basis of the notion that protein stability generally promotes evolvability,^61^ it has further been proposed that ancestral enzymes represent ideal scaffolds for further sequence optimization,^13^ thus being the starting and not the ending point of enzyme engineering programs. Such studies have involved, e.g., DNA-shuffling of ambiguous ancestral residues in a cytochrome P450 to further screen for enhanced thermostability and developing an ancestral amino acid binding protein into a biotechnologically applicable arginine biosensor.^13,62^

Crystal structure guided targeted modification of ancestral enzymes has less commonly been explored. One notable example includes the design of a de novo Kemp eliminase activity by a rational single amino acid substitution in ancestral β-lactamase scaffolds.^63^

Considering the thermal stability of SvS-A2 combined with the derived knowledge of substrate positioning in the active site cavity and the defined role of a limited number of key residues, we assumed that the ancestral enzyme could generally function as an evolvable scaffold to enhance specificity and activity. We further hypothesized that ancestral-structure informed engineering could represent a viable approach for redesigning the extant terpene cyclase as well. This hypothesis is supported by the fact that the fold and active site architecture of ancestral and extant SvS are essentially conserved (Figure S8a, Tables S3 and S7) and that their overall activities are comparable (Figure 3).

To this aim, a small library of 24 variants of SvS-A2 was rationally designed, based on the SvS-A2 crystal structure targeting the active site as well as surrounding residues (Figure S8a, Table S7). Most residues that are suggested to be involved in carbocation stabilization from mechanistic considerations above were not targeted (such as Phe59 or Trp308) in order to avoid creating inactive enzymes and to maintain a largely hydrophobic active site. Instead, we focused on changing the size of residues that are adjacent to carbocation-stabilizing residues to modulate the steric access in the active site cavity. Such changes include, e.g., Val80Ile, which was designed to close the bottom of the active site cavity with a larger hydrophobic residue (Figure 4, panels **1** and **2**) or Gly301Phe that adds a bulkier hydrophobic residue in proximity of Trp308 (Figure 4, panel **3**). Furthermore, the size of residues in the vicinity of the effector motif was modified in order to make the active site larger or smaller (such as, e.g., Leu183Trp, Ala186Gly, and Gly188Leu, Figure 4, panels **4** and **6**).

The first carbocation generated (Figure 4, panel **2**) is located close to the lower part of the NSE motif, and the size of residues in proximity of this motif were changed in order to restrict access (e.g., Ala224Ile or Ala225Phe).

A few residues among the selected positions were not conserved between SvS-WT and SvS-A2 and were changed to the respective wild-type residue in the ancestral background (e.g., Val127Ile, Thr191Pro, Leu277Met, Table S7). Several positions were also changed to introduce the equivalent residues from SdS (Table S3), which was used as a search model for molecular replacement.

The catalytic DDxx(x)D metal binding motif of SvS-WT was retained in all library variants, involving an Ala89His exchange. In this way, any eventual effects from an altered metal binding site are excluded, since this is the only position within the otherwise conserved catalytic motifs that differs between SvS-A2 (Ala89) and SvS-WT (His89). The effect of this exchange on enzyme performance and specificity was previously shown to be minor in SvS-A2.^26^ The 205–209:DREMH/AQDLE surface variant of SvS-A2 that was used for construction of the improved homology model was included for comparison.

**Figure 5:**
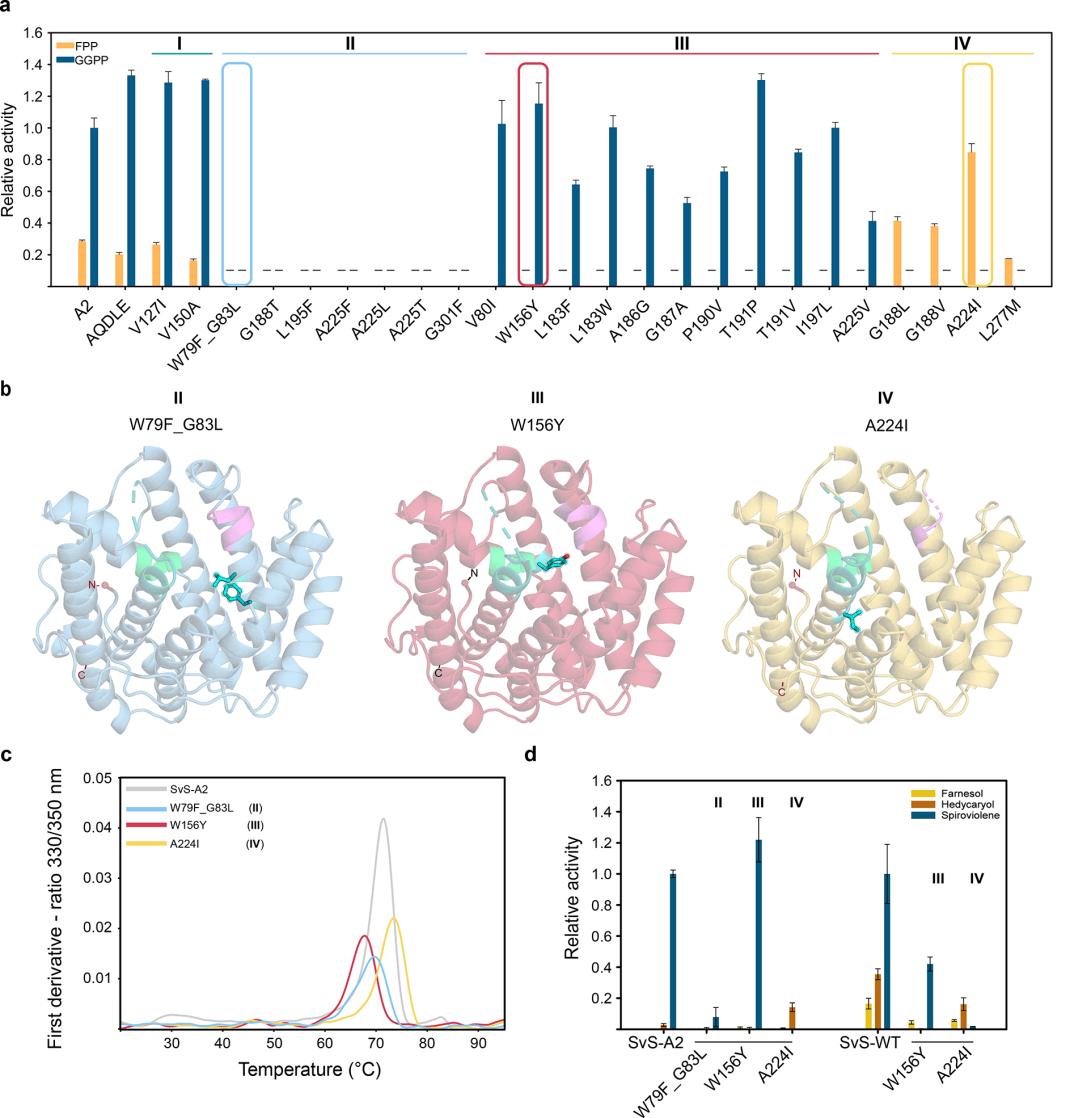
Rational enzyme engineering of ancestral terpene cyclase SvS-A2. (a) Activity of variant library based on SvS-A2 as scaffold measured by Malachite Green assay using single substrates. Variants are classified as inactive (I), promiscuous (II), GGPP-specific (III), or FPP-specific (IV). Activities are given relative to SvS-A2 GGPP activity (defined as 1.0). Variants with values below the sensitivity threshold are represented with a dash. Error bars are standard deviations from triplicates. Selected representative variants for each group are enclosed by colored boxes. (b) Crystal structures of the three representative variants from (a) with the mutated residues shown as cyan sticks and balls. The DDxx(x)D motif is shown in violet, NSE motif in cyan/teal, effector motif in light green. (c) Thermal stability of representative variants of SvS-A2. Melting temperatures were determined by nano-DSF as the maximum of the derivative of the 330/350 nm ratio (technical triplicates, one representative trace is shown). (d) Product formation by representative variants assessed by GC-FID. Triplicates using ca. 400 nM of SvS-A2 variants and 2 μM of SvS-WT variants were incubated with a mix of both 60 μM FPP and GGPP (3 h at 30 °C) in vitro (different enzyme concentration used due to different protein stability). Product formation was quantified relative to an internal standard. Activities are given relative to SvS-A2 GGPP activity (for SvS-A2 variants) and relative to SvS-WT GGPP activity (for SvS-WT variants, each defined as 1.0, respectively).

The variants could be grouped into four categories based on their substrate preference in a Malachite Green assay: promiscuous (I), inactive (II), diterpene-specific (III), or sesquiterpene-specific (IV) (Figure 5a). One representative variant of each group was subjected to crystal structure analysis (Figure 5b, Table 1), and Cα rmsd values in the range of 0.35–0.68 Å compared to the parental SvS-A2 structure indicated that none of the variants had significant conformational rearrangements. Therefore, the altered substrate preferences are likely the consequence of steric and/or electronic interactions with substrates, intermediates and/or products, as discussed for the individual variants below. Furthermore, the studied variants were dimeric in the crystal structure and maintained a substantially elevated melting temperature similar to that of SvS-A2 (Figure 5c). Substrate specificity of one representative of each group with altered activity over SvS-A2 (groups II–IV) was verified in a GC flame ionization detection (GC-FID) competition experiment (Figure 5d, left), qualitatively confirming the results from the Malachite Green assay.

The low diterpene activity of Trp79Phe_Gly83Leu (group II) is expected, as Trp79 is involved in stabilization of the first carbocation (intermediate **2** in Figure 4). The Gly83Leu substitution may shield off not only Phe79, but also Phe82 and Phe84 from the substrate, which are putatively relevant for cation stabilization. Since our modeling data suggest that Phe84, which is conserved in bacterial sesquiterpene cyclases,^31^ is involved in stabilizing cations for both the sesquiterpene and diterpene reactions (Table S3), sterically blocking this residue by the Gly83Leu exchange results in a near inactive protein (Figure 5a).

A Trp156Tyr exchange (Group III) results in a highly active and diterpene specific SvS-A2 variant (Figure 5a). The variant’s specificity for the formation of the diterpene spiroviolene over the formation of sesquiterpenes hedycaryol and farnesol can be expressed as the ratio of apparent second order rate constants and increases ca. 2.3-fold from 38.4 ± 13.7 in SvS-A2 to 88.1 ± 29.8 in the Trp156Tyr variant of SvS-A2. The exchanged position is located directly opposite of Trp79 and the introduction of Tyr may allow for additional cation-π interactions in **2**, while yielding sufficient space to accommodate the bulky diterpene intermediate between Trp79 and Trp82. Alternatively, the introduced hydroxyl group may allow hydrogen bonding with residues in the adjacent effector motif.

The single residue exchange Ala224Ile (group IV) reverses substrate preference of the reconstructed ancestral enzyme and enhances specific activity for FPP approximately 3–5-fold (Malachite Green assay/GC-FID) compared to the reconstructed ancestral enzyme (Figures 5a,d). Spiroviolene formation could not be detected for this variant so that a ratio of apparent second order rate constants cannot be derived. The isoleucine in this position is conserved in the two bacterial sesquiterpene cyclases SdS and HecS (Ile220 and Ile217, respectively, Table S3),^31,51^ indicating that it may be a specificity switch of general importance in directing bacterial terpene cyclase sesquiterpene specificity. Changing this residue might affect metal binding as it is located upstream of the NSE motif. Steric effects are also likely to play a role (Figure S8b,c), since the larger size of an isoleucine side chain permits sesquiterpene binding while hindering the correct positioning of the bulky diterpene substrate in the active site.

We wondered if the ancestral background is required for harboring the identified reciprocal specificity switches; Trp156Tyr for GGPP-specificity and Ala224Ile for FPP-specificity, respectively. Introducing the corresponding single residue exchanges into the SvS-WT sequence resulted in variants with similar switch of specificity, albeit with overall lower activity than the wild type (Figure 5d, right). Specificity for formation of spiroviolene over formation of hedycaryol and farnesol increased ca. 4.4-fold (from 2.1 ± 0.2 in SvS-WT to 9.1 ± 1.7) in the Trp156Tyr variant and decreased ca. 25.4-fold to 0.08 ± 0.02 in the Ala224Ile variant. Moreover, the contribution of these residue exchanges to thermostability appears to follow a similar trend as in the ancestral enzyme (Figure 5C, Figure S9). The Trp156Tyr exchange slightly reduces the melting temperature of SvS-A2 (from 71.3 ± 0.0 °C to 67.7 ± 0.1 °C) and more so of SvS-WT (from 58.2 ± 0.1 °C to 45.2 ± 0.3 °C). In contrast, the Ala224Ile exchange slightly increases both the melting temperature of SvS-A2 (from 71.3 ± 0.0 °C to 73.4 ± 0.1 °C) and of SvS-WT (from 58.2 ± 0.1 °C to 63.6 ± 0.1 °C). The present study highlights how substrate specificity can be controlled in an ancestral terpene cyclase scaffold in a structure-guided targeted manner, without compromising protein stability. The combination of mutations that together enable specificity and stability in the ancestor would likely have been difficult to identify starting from the extant enzyme.

A previous study that generated a de novo active site in an ancestral β-lactamase showed that introducing the equivalent substitutions in extant homologues did not yield the novel activity.^63^ We show how single residue exchanges that confer substrate specificity in an ancestral terpene cyclase can be transferred to the extant enzyme, affording an analogous specificity shift. While a previous study has pinpointed the determinants of specific protein interactions by transferring the ancestral mutations to the extant enzyme,^64^ we find that this transfer can also work with residues that are originally neither present in the ancestor, nor the extant enzyme. The Ala224Ile exchange reverses substrate preference, and due to its conservation in a functionally related sesquiterpene cyclase, we speculate that it may represent a general specificity switch in bacterial terpene cyclases.

## Conclusion

Natural products have found important applications as medicines, renewable chemical building blocks, and polymer precursors. Roughly half of all FDA-approved drugs are based on natural products or derivatives thereof, and understanding how nature assembles its array of chiral, complex structures from simpler metabolites is a long-standing goal in enzymology and synthetic biology.^2,3,65,66^ However, one bottleneck preventing the full application potential of X-ray crystallography is the limited solubility and stability displayed by some proteins.^17^

We hypothesized that inferred ancestors, due to inherently high sequence identities and presumed homology, represent potent templates for constructing high-confidence homology models of extant enzymes that are not prone to crystallization. We have critically evaluated this notion and show that further engineering was required to afford an improved homology model of an extant class I terpene cyclase.

Redesigning the surface of the ancestral enzyme to restore wild-type sequence at selected positions provided a crystal structure representing an optimized template and improved the confidence in the derived homology model of the extant enzyme, in particular for regions involved in substrate and cofactor binding. We therefore reason that critical assessment of ancestral mutations in conjunction with the obtained structural information can yield more reliable templates for studying complex extant enzymes and their reaction mechanisms.

The workflow of generating a robust ancestral enzyme from an alignment of extant sequences, crystallizing it, and using the information for structure-guided engineering of specificity in ancestral and extant enzymes, resulted in specific and active terpene cyclase variants and identification of key residue exchanges that control substrate specificity.

In summary, our results demonstrate the utility of reconstructed ancestors in structural biology in order to study extant biosynthetic enzymes that are challenging to crystallize. We anticipate that the approach herein will be useful in unravelling structures of other metabolic enzymes, allowing to understand their reaction mechanisms and enabling to engineer their remarkable catalytic versatility.

## Supporting Information Available

Materials and Methods, Figures S1–S9, Tables S1–S7, and Supporting References (PDF)
