# Supplementary material for: Engineering of Ancestors as a Tool to Elucidate Structure, Mechanism, and Specificity of Extant Terpene Cyclase
Source: J Am Chem Soc. 2021 Jan 26;143(10):3794–807. doi: 10.1021/jacs.0c10214 (PMC8023661; doi:10.1021/jacs.0c10214)
Supplement: Supplementary file 1 — ja0c10214_si_001.pdf [file ja0c10214_si_001.pdf]

Engineering of Ancestor as a Tool to Elucidate Structure, Mechanism and Specificity of Extant Terpene Cyclase

Karen Schriever,[#,‡] Patricia Saenz-Mendez,[#,‡] Reshma Srilakshmi Rudraraju,[¶] Natalie M. Hendrikse,[#,‡,^] Elton P. Hudson,[#,°] Antonino Biundo,[#,‡,†] Robert Schnell,[¶]* Per-Olof Syrén[#,‡,Δ]*

[#] School of Engineering Sciences in Chemistry, Biotechnology and Health, Science for Life Laboratory, KTH Royal Institute of Technology, Stockholm, Sweden.

[‡] School of Engineering Sciences in Chemistry, Biotechnology and Health, Department of Fibre and Polymer Technology, KTH Royal Institute of Technology, Stockholm, Sweden.

[¶] Department of Medical Biochemistry and Biophysics, Karolinska Institutet, S-17 165 Stockholm, Sweden.

^ Swedish Orphan Biovitrum AB, Stockholm, Sweden.

° School of Engineering Sciences in Chemistry, Biotechnology and Health, Department of Protein Science, KTH Royal Institute of Technology, Stockholm, Sweden.

[Δ] Wallenberg Wood Science Center, Teknikringen 56–58, 100 44 Stockholm, Sweden.

Supporting Information

Table of Contents

Materials and Methods. **pp. S2-7**

Supporting Figures.
S1. Ancestral sequence reconstruction of SvS. **p. S8**
S2. Stability and solubility of extant and reconstructed ancestral SvS. **p. S9**
S3. Comparison of the different homology models of SvS-WT. **p. S12**
S4. Characterization of SvS-A2 products. **p. S15**
S5. Thermal analysis of elemol formation. **p. S16**
S6. Alternative penultimate intermediate in the GGPP cyclization cascade. **p. S19**
S7. Snapshots of the SvS-A2 catalyzed cyclization of FPP to hedycaryol. **p. S20**
S8. Overview of SvS-A2 variant library. **p. S22**
S9. Thermal stability of specificity switch variants of SvS-WT **p. S24**

Supporting Tables.
S1. Crystallization Conditions, Crystal Treatment and General Structure Attributes. **p. S10**
S2. Modeling Scores Obtained from YASARA upon Homology Modeling of SvS-WT. **p. S11**
S3. Active Site Conservation Between SvS-WT, SvS-A2, Hedycaryol Synthase and Selinadiene Synthase. **pp. S13-14**
S4. Calculations of Binding Energies Between Monomeric Holoenzymes and Either GGPP or FPP. **p. S17**
S5. Key Atom Distances in Intermediates along the Reaction Pathway in the Formation of Spiroviolene. **p. S18**
S6. Key Atom Distances in Intermediates along the Reaction Pathway in the Formation of Hedycaryol. **p. S21**
S7. Brief Description of SvS-A2 Variant Library. **p. S23**

Supporting References. **pp. S25-26**

Materials and Methods

Unless stated otherwise, chemicals were purchased via Sigma Aldrich Sweden. Enzymatic substrates farnesyl pyrophosphate (FPP) and geranylgeranyl pyrophosphate (GGPP) were purchased as ammonium salts (≥95% thin layer chromatography). Hexane used as solvent for gas chromatographic analysis was purchased in analytical reagent grade from Fischer Scientific, United Kingdom.

Ancestral Sequence Reconstruction

Ancestral sequence reconstruction was performed previously.[1] Briefly, we used maximum likelihood statistics implemented in MEGA7 to reconstruct ancestral SvS enzymes, utilizing extant spiroviolene synthase (SvS) from *Streptomyces violens* (referred to as SvS-WT in this study) as query for basic local alignment search and sequence alignment. The ancestral enzyme used as model system herein is referred to as SvS-A2 (sequence information for SvS-WT and SvS-A2 given in Figure S1).

Generation of Enzyme Variants and Protein Expression

Genes used in this study comprise SvS-WT and SvS-A2 (sequences for both given in Figure S1) and a surface variant of SvS-A2 (205-209:DREMH/AQDLE) with an additional Ala89His exchange. Variants of SvS-A2 are described in Table S7 and variants of SvS-WT comprise a Trp156Tyr and Ala224Ile exchange.

The genes of interest were purchased from GeneArt Services (Thermo Fischer Scientific, USA) as codon-optimized sequences with an N-terminal His_6-tag, cloned into the expression vector pET22b(+), and transformed into chemically competent *Escherichia coli* BL21(DE3). Cells were plated on Lysogeny Broth (LB)-Agar plates supplemented with 100 µg mL^{-1} ampicillin and incubated overnight at 37 °C. Individual colonies were used to inoculate 5-10 mL LB medium (100 µg mL^{-1} ampicillin freshly added) and were grown overnight at 37 °C and 200 rpm. Cells were propagated by inoculating 2xYT medium (16 g L^{-1} tryptone, 10 g L^{-1} yeast extract, 5 g L^{-1} NaCl, 100 µg mL^{-1} ampicillin freshly added) with overnight cultures to an optical density at 600 nm (OD_{600}) of 0.05 and were further grown to an OD_{600} of 0.6-0.8 at 37 °C and 200 rpm. Cells were subsequently transferred to 18 °C, 160 rpm for 15 min and induced with 0.4 mM isopropyl β-D-1-thiogalactopyranoside for 22 h at 18 °C, 160 rpm. Cells were harvested by a 20 min centrifugation (4 °C, 3800 x *g*), the supernatant was discarded and cell pellets were stored at -20 °C.

Protein Purification

Cell pellets were lysed with B-PER Complete Bacterial Protein Extraction Reagent (Thermo Fischer Scientific, USA, 5 mL g^{-1} wet cell pellet weight) with 20 mM imidazole for 15 min at 25 °C, 80 rpm in a cultivation shaker. The resulting lysates were cleared by centrifugation (4 °C, 20 min, 3800 x *g* for small expression volumes for in vitro reactions or 18.000 x *g* for larger expression volumes for crystallization) and filtered through a 0.2 µm syringe filter (VWR Europe). Purification was performed by immobilized nickel ion affinity chromatography using 1 mL HisTrap™ HP-columns in an ÄKTA Start chromatography system (GE Healthcare Life Sciences, Sweden) with a flowrate of 1 mL min^{-1}. For preparation of proteins for crystallization 5 mL columns were used. Lysate was applied to columns that were pre-equilibrated with wash buffer (50 mM tris(hydroxymethyl)aminomethane (Tris)-HCl, 500 mM NaCl, 20 mM imidazole, pH 7.4 at 25 °C) and washed with 15 column volumes of wash buffer. Elution was performed using a stepwise gradient of elution buffer (50 mM Tris-HCl, 500 mM NaCl, 300 mM imidazole, pH 7.4 at 25 °C). The presence of target protein was assessed by sodium dodecyl sulfate polyacrylamide gel electrophoresis (Bio-Rad Laboratories, USA). For in vitro activity assays pooled protein fractions were desalted into 50 mM Tris-HCl, pH 7.4 at 25 °C by gravity flow using PD-10 desalting columns packed with Sephadex G-25 (GE Healthcare Life Sciences, Sweden).

Protein preparations for crystallography were subjected to size exclusion chromatography using a HiLoad 16/600 Superdex 75 prep grade column (GE Healthcare Life Sciences, Sweden) on an ÄKTA Start chromatography system at a flow rate of 1 mL min^{-1}, using a 25 mM Tris-HCl, 150 mM NaCl, pH 8.0 (at 25 °C) buffer.

Pure protein fractions were concentrated with centrifugal protein filters (Vivaspin 20 mL, 10 kDa molecular weight cutoff, Sartorius, Germany) that were pre-equilibrated with the respective desalting buffer at 3800 x *g*, 4 °C. Protein concentrations were determined by spectrophotometry at 280 nm on an Implen NanoPhotometer NP80 (Germany) using calculated molar extinction factors. For crystallization experiments, protein concentrations were further verified by the Bradford assay using a commercial reagent (Thermo Fischer Scientific, USA).

For purification of the variant library, a 96-well plate-based centrifugation protocol was used. In specific, cell pellets from 5 mL cultivation volume were resuspended in 600 µl B-PER Complete Bacterial Protein Extraction Reagent supplemented with 20 mM imidazole and incubated for 15 min at room temperature on a plate shaker (750 rpm). Lysates were cleared by 20 min centrifugation (4 °C, 2300 x *g*) and transferred to individual wells of a His MultiTrap Fast Flow plate (GE Healthcare Life Sciences, Sweden) that was previously equilibrated with wash buffer. The plates were incubated with the lysate for 20 min at room temperature on a plate shaker (250 rpm) and the flowthrough was removed by 4 min centrifugation (4 °C, 100 x *g*). Washing was performed twice by adding 500 µl of wash buffer and removing wash-fractions by centrifugation (500 x *g*, 2 min, 4 °C). Proteins were eluted by adding 150 µl of elution buffer and incubating the plates at room temperature for 3 min. Elution fractions were then collected in a fresh collection plate by centrifugation (500 x *g*, 2 min, 4 °C). The eluate was transferred to a PD MultiTrap G-25 plate (GE Healthcare Life Sciences, Sweden) that was pre-equilibrated with desalting buffer (50 mM Tris-HCl, pH 7.4 at 25 °C). Desalted fractions were obtained by adding 50 µl of desalting buffer to the samples and collecting the eluate in a fresh collection plate by centrifugation (1 min, 800 x *g*, 4 °C). Protein concentrations were determined spectrophotometrically at 280 nm as described above.

All protein preparations were kept at 4 °C upon purification until used for activity assays or flash-frozen in liquid nitrogen for crystallographic studies.

Thermostability Measurements

Thermostability measurements were performed by nano differential scanning fluorimetry (nanoDSF) on a Prometheus NT.48 nanoDSF instrument (NanoTemper Technologies, Germany). Desalted protein solutions (20 µM for SvS-A2 and SvS-WT, 4-6 µM for selected variants) were soaked into a glass capillary and thermal unfolding was recorded from 20 °C to 95 °C at a rate of 1 °C min^{-1} (excitation power of 25%) by monitoring the ratio of protein autofluorescence at 330 nm and 350 nm. Melting points were determined as the point equivalent to the maximum of the first derivative of the change of the 330 °C / 350 °C ratio.

Size Exclusion Chromatography with Multi Angle Light Scattering detection (SEC-MALS)

Purified and desalted enzymes (50 µl of 3-8 mg mL^{-1}) were analyzed by size exclusion chromatography on a Superdex 200 Increase 10/300 GL column (GE Healthcare, Sweden) in an ÄKTAmicro chromatography system (GE Healthcare, Sweden) at a flow rate of 0.75 mL min^{-1} using filtered Phosphate Buffered Saline (pH 7.4) for elution. The chromatography system was coupled to a miniDAWN multi angle light scattering detector (Wyatt Technology, USA) for molecular weight detection.

The elution profile was determined based on the differential refractive index and molecular weights were determined using ASTRA 5.3.4.20 software (Wyatt Technology, USA).

Protein Structure Determination

The crystallization screens were carried out at 14.6 mg mL^{-1} protein concentration using a Mosquito crystallization robot and commercial screens (PACT, JSGC+ obtained from Qiagen and Wizard screen from Emerald) in sitting drop format by the vapor diffusion method. For crystal production the hanging drop setup was employed in 24-well cell culture plates (Sarstedt, Germany). Rod shaped crystals of SvS-A2 and derived variants were obtained using 0.1 M Bis-Tris-Propane, 0.2 M Na-phosphate 20% (v/v) PEG 3350, pH 6.5. The design variants of SvS-A2 were crystallized in similar conditions with variation of pH 6.3-7.2 and PEG3350 concentrations from 12% to 20%. Further details and cryo-protection of the protein crystals are provided in Table S1. Cocrystallization was attempted with the substrates FPP and GGPP by adding the substrates at 5 mM and $MgCl_2$ at 5 mM concentration to the protein solution 30 min prior to setting up the crystallization drops and at the cryoprotection stage (Table S1). The X-ray diffraction datasets with the best resolution in the cases of Trp79Phe_Gly83Leu, Ala224Ile and Trp156Tyr variants are from these cocrystallization attempts with FPP, however no ligand complex was obtained.

The X-ray diffraction dataset to 2.3 Å resolution for SvS-A2 was collected at beamline ID23-1 of the European Synchrotron Radiation Facility (ESRF, Grenoble, France) at 100 K. The X-ray diffraction datasets for SvS-A2 variants Trp79F_Gly83Leu, Trp156Tyr, Ala224Ile and 205-209:DREMH/AQDLE to 2.27-2.6 Å were collected at the BIOMAX beamline of MAX-IV (Lund, Sweden) at 100 K. The X-ray data were indexed and processed with XDS[2] and subsequently scaled with AIMLESS from the CCP4i suite.[3] The crystals belong to space group $P2_12_12_1$ with cell dimensions a = 75.3 Å, b = 105.5 Å, and c = 105.5 Å for SvS-A2 with minor variation for the design variants. The statistics of the data sets are given in Table 1.

The structure of the reconstructed ancestral protein SvS-A2 was solved by molecular replacement using MOLREP[4] and the poly-Alanine model derived from the coordinates of selinadiene synthase (*SdS*, PDB-ID: 4OKZ[5]), a bacterial class I sesquiterpene cyclase from *Streptomyces pristinaespiralis*.

The model was built and refined by employing Arp/wArp[6] and continued with manual model building using COOT[7] intervened by cycles of restrained refinement using REFMAC-5.[8] Water molecules were placed by COOT and subsequently checked by manual inspection of the molecular contacts and electron density. The final model contains a dimer of the terpene synthase all α-fold representing amino acid residues 7-357 in chain-A and 8-357 in chain-B, and 274 water molecules with final crystallographic R and Rfree values of 0.185 and 0.219, respectively. Disordered surface loops lacking electron density prevented the modeling of the residue ranges 232-238 and 310-326 chain-A; and the residue ranges 88-95, 232-241 and 311-326 in chain-B (Table S1). The structures of the designed variants (Table 1, Table S1) were solved by using the SvS-A2 structure as search model and refined using a similar protocol as for SvS-A2, leading to the 2.27 Å structure of Trp79Phe_Gly83Leu, the 2.38 Å structure of 205-209:DREMH/AQDLE, the 2.40 Å structure of Trp156Tyr and to the 2.60 Å structure of Ala224Ile variants of SvS-A2 presenting approximately the same disordered residue ranges as the parental SvS-A2 species (Table S1). Macromolecular interfaces and solvent accessible surfaces were analyzed at the PISA server.[9] The protein models were validated with regard to stereochemistry and model quality using COOT and MOLPROBITY.[10] Statistics from the refinement and model quality are provided in Table 1, the final models and structure factors are deposited with the Protein Data Bank with accession codes 6TBD, 6TJA, 6TIV, 6THU and 6TJZ.

Construction of Missing Loops and Metal Ion Cluster for SvS-A2 and SvS-A2 Surface Variant

The crystal structure of SvS-A2 has unresolved residues, thus preventing proper description of the 3D structure of the entire system. Mg^{2+} ions were included in several crystallization and soaking experiments, yet no metal ions were found in the crystal structures. To obtain a complete protein structure, the full amino acid sequence of SvS-A2 was employed to build a model of missing loops and the protein termini using YASARA.[11-12] Default parameters were employed for modeling of the loops and termini. Hydrogen atoms were added to the structure according to the pK_a of residues and a pH of 7. Side chains for the unresolved parts were built and the loop structures were optimized by exploring a large number of conformations using YASARA rotamer libraries to generate an initial structure. Subsequently, side chains and loops were subjected to a combined steepest descent and simulated annealing minimization, keeping backbone atoms fixed. Finally, a fully unrestrained simulated annealing minimization was performed on the entire system. Convergence in minimization stages was reached when the energy improved less than 0.05 kJ mol^{-1} during consecutive steps. All refinement steps were conducted using a knowledge-based force field included in YASARA (YASARA2 force field) which has been optimized for structure prediction, refinement, energy minimization and validation.[11] The tri-Mg^{2+} ion cluster was built into the minimized structure by superposition onto SdS (PDB-ID: 4OKZ[5], Cα root mean square deviation (rmsd) of 1.26 Å over 312 residues). Lastly, the structure was minimized using the same force field as above and docked to the substrate GGPP (described in detail below). Missing loops in the SvS-A2 surface variant were generated in YASARA in the same way; the metal ion cluster and substrate were included from superposition with the final docked SvS-A2 model. Figures were generated using The PyMOL Molecular Graphics System, version 2.3.4 Schrödinger, LLC.

Molecular Docking

In order to obtain a refined model of SvS-A2 (full model including loops and metal ions) complexed to the substrate, GGPP was docked into the active site of monomeric SvS-A2 using the Molecular Operating Environment (MOE) 2015.10.[13] All described energy minimizations were performed using the AMBER10 force field to within an RMS gradient of 0.01 kcal mol^{-1} $Å^{-1}$ and using a dielectric constant of three for the protein and an external dielectric of 80 to simulate an implicit water environment.[14]

GGPP was constructed and energy minimized. Five hundred poses were retained for each ligand using the alpha-triangle placement methodology with binding affinity (ΔG) as scoring function as embedded in MOE. In the docking experiments, flexible ligand structures were generated using a Monte Carlo algorithm, while the protein was held fixed at its energy-minimized geometry. The top scored orientations of the products were then energy minimized, followed by energy minimization of the entire system. Since no productive prefolded conformation of GGPP could be obtained in this automated way, the product spiroviolene was built and successfully docked using the same settings as described. Subsequently, the experimentally suggested reaction mechanism for spiroviolene formation was followed in the backward direction to obtain a productive starting conformation of

GGPP.[15] To this end, bonds were manually broken and formed to generate the suggested intermediates and finally GGPP. For each generated intermediate (and GGPP) energy minimization was performed, keeping the ligand and any protein atoms within a radius of 8 Å around the ligand fully flexible. Then the entire system was energy minimized. The intermediates were also generated by breaking and making bonds going in the forward direction, starting from productively prefolded substrate. This process was repeated iteratively until the conformation of intermediates converged when following the reaction in forward and backward direction. The process was repeated following the suggested intermediates in the formation of hedycaryol (based on the mechanism of another bacterial hedycaryol synthase)[16], starting by superpositioning SvS onto PDB-ID: 4MC3 to generate initial coordinates of the substrate. For analysis of water as base/nucleophile, the entire system was placed in an explicit water box during minimization for spiroviolene **8**, hedycaryol **14** and their preceding cations **7** and **13**. Water molecules with a radius of up to 9 Å around the ligand were kept for further analysis.

Substrate Binding Affinity (Δ*G*)

The monomeric SvS-substrate complexes were refined by a final energy minimization (RMS gradient of 0.01 kcal mol^{-1} $Å^{-1}$) using the LigX interface in MOE. The protein atoms far from the substrate were kept fixed, while protein atoms in the binding site (defined at a distance of 8 Å from the substrate) were treated as fully flexible. This allows to account for protein flexibility, that is induced fit. After refinement, the binding affinity was calculated employing the GBVI/WSA Δ*G* scoring function.

Homology Modeling of SvS-WT

Different templates were used to build homology models of the extant enzyme: (i) the obtained crystal structure of SvS-A2 (PDB-ID: 6TBD) including modeled loops and the Mg^{2+} cluster docked to GGPP (SvS-WT-Hom1) (ii) an engineered surface variant of SvS-A2 (PDB-ID: 6TIV) including modeled loops and GGPP as well as the Mg^{2+} cluster (SvS-WT-Hom2) (iii) SdS, the enzyme (PDB-ID: 4OKZ) in the PDB with highest sequence identity to SvS, including fully resolved loops as well as Mg^{2+} cluster bound to a substrate analog (SvS-WT-Hom3). Templates provided during the homology modeling process were monomeric since accurate substrate-bound models obtained from the docking experiments were monomeric. Parameters for construction of the homology models in YASARA were set to default values, while setting the maximum number of templates to be used to one and providing the respective input template structure as PDB file. Confidence in the model was primarily assessed using the obtained Z-score for dihedral angles. The quality of the models was further assessed by Ramachandran plot analysis (RAMPAGE server), Verify 3D analysis and ERRAT 3D-1D profile.[17-22] Generation and manual docking of intermediates and substrates to the final homology model (SvS-WT-Hom2) was performed as described above for SvS-A2.

Product Characterization

The major products of enzymatic conversion were identified by mass spectrometry (MS) and flame ionization detection (FID) analysis on a dual detector GCMS-QP2010 Ultra (Shimadzu, Japan), using an Rxi-5ms capillary column (30 m length, 0.25 µm thickness, 0.25 mm i.d., Restek, USA). Purified and desalted SvS-A2 protein (2 µM) was incubated for 180 min in 2 mL capped glass vials (30 °C, 1200 rpm in Thermomixer (Eppendorf, Germany)) with 60 µM of either FPP or GGPP in reaction buffer (50 mM Tris-HCl, 1 mM $MgCl_2$, pH 7.4 at 25 °C) in a total reaction volume of 200 µl. Reaction products were immediately extracted by adding 2x 300 µl hexane with a Hamilton syringe, vortexing and centrifuging (room temperature, 9000 x *g*, 10 min) and transferring the total amount of solvent phase to a GC-vial with a glass Pasteur pipette. For MS-characterization, 1 µl of the solvent phase was injected at 150 °C or 250 °C with a split ratio of 1.5. The column oven temperature was increased from 50 °C to 250 °C at a rate of 8 °C min^{-1} and then further raised to 330 °C at a rate of 15 °C min^{-1} and kept at 330 °C for 5 min under a pressure controlled flow of helium (100.0 kPa). Electron ionization was performed at an ion source temperature and transfer line temperature of 200 °C and a full scan of *m*/*z*-values ranging from 20 to 400 was recorded. Products were identified by comparing the resulting spectra to spectra in published literature and the NIST library version 11/11s. For identification of farnesol a (2*E*,6*E*)-farnesol standard was used.

Linear retention indices (Van den Dool- and Kratz-indices) were determined to identify farnesol, hedycaryol and spiroviolene by GC-FID analysis of extracts spiked with an analytical alkane standard (C_8 – C_{20}).[23] To this end, 1 µl of spiked extract was injected at 250 °C with a split ratio of 10.0. The column oven temperature was programmed based on a protocol published by Baer et al.[16]: the temperature was kept at 50 °C for 5 min and then raised to 320 °C at a rate of 10 °C min^{-1} and kept at 320 °C for 3 min under a pressure controlled flow of helium (160.9 kPa). The FID-detector was set to 350 °C using a makeup flow of 30 mL min^{-1} helium. Linear retention indices were

then calculated based on relative retention of target compounds in reference to the two adjacent alkane peaks. The identity of hedycaryol was finally confirmed by analysis of thermal rearrangement to elemol[24]. The injection temperature was 150 °C, spectra were recorded using both full scans and selected ion monitoring (*m*/*z* values of 59, 93 and 161).

Product Quantification by GC-FID

Products were quantified by comparing GC-FID peak areas to the peak area of an internal decane standard and accounting for estimated relative response factors (GC instrumentation described above).[25] To this end 1 µl of extracted solution was injected at 250 °C with a split ratio of 10.0. The column oven temperature was increased from 50 °C to 250 °C at a rate of 8 °C min^{-1} and then further raised to 330 °C at a rate of 15 °C min^{-1} and kept at 330 °C for 5 min under a pressure controlled flow of helium (160.9 kPa). The FID detector was set to 350 °C using a makeup flow of 30 mL min^{-1} of helium.

Enzyme Kinetics

For kinetics of the reconstructed ancestral enzyme with either FPP or GGPP, experiments were performed in triplicate. Substrate concentrations ranging from 5 to 120 µM were prepared using a substrate stock solution (in 70% methanol/30% aqueous ammonia) in 2 mL capped glass vials in a total volume of 410 µl reaction buffer (50 mM Tris-HCl, 1 mM $MgCl_2$, pH 7.4 at 25 °C). In all diluted substrate solutions the methanol concentration was adjusted to the maximum volume resulting from addition of methanol containing substrate stock within the experiment (corresponding to 4.2% in the reaction vials). The substrate dilutions were pre-incubated for 10 min at 30 °C in a thermomixer (Eppendorf, Germany) and the temperature of the thermomixer was verified with an external thermometer. Then, 10 µl of purified and desalted enzyme were added to each vial to reach a final enzyme concentration of 500 nM. After mixing by pipetting, 100 µl of solution were immediately withdrawn, transferred to a glass vial and extracted with 2 x 150 µl of hexane spiked with 10 µM decane as described above. After withdrawing the initial 100 µl aliquot, the reaction was incubated at 1200 rpm shaking speed and 100 µl aliquots were withdrawn after 30, 60 and 90 min of incubation and extracted following the same protocol. Product concentrations in all extracts were quantified by GC-FID as described above and initial rates (stated in nanomoles per milligram per minute) were calculated from the slope of product accumulation over time (linear over the entire duration of the incubation). Kinetic parameters were obtained by fitting the observed initial rates to the Michaelis-Menten and Hill-equation using nonlinear regression in the Enzyme Kinetics tool in SigmaPlot version 14.0:

$$v_0 = \frac{v_{\mathrm{max}} \times [\mathrm{S}]^h}{{K_{\mathrm{M}}}^h + [\mathrm{S}]^h} \qquad \textit{Eq.1}$$

where v_0 and v_{max} are initial and maximal rates, K_M is the Michaelis-Menten constant, [S] is the concentration of substrate and *h* is the hill coefficient (for regular Michaelis-Menten kinetics *h* is 1.0).

Relative Diterpene Specificity

Relative diterpene specificity was determined in triplicates based on the accumulation of the major products (spiroviolene and hedycaryol) in an in vitro competition assay. An equimolar substrate mix of FPP and GGPP (60 µM each) was prepared in a total volume of 95 µl of reaction buffer (50 mM Tris-HCl, pH 7.4, 1 mM $MgCl_2$) in 2 mL capped glass vials. The substrate concentration of 60 µM was chosen to achieve maximum detection signal on the GC-FID detector while ensuring that the enzyme was not saturated. The substrate dilutions were pre-incubated for 10 min at 30 °C in a thermomixer (Eppendorf, Germany) and the reaction was started by adding 5 µl of purified and desalted enzyme solution, resulting in a final enzyme concentration of 0.5 or 2 µM. The reactions were incubated at 1200 rpm, 30 °C for 180 min and then extracted as described above. Reaction products were quantified by GC-FID as described above and normalized by the enzyme concentration. Diterpene specificity was expressed as ratio of apparent second order rate constants of the major diterpene product (spiroviolene) over the major sesquiterpene product (hedycaryol) based on the following equation:

$$\frac{(k_{\mathrm{cat}}/K_{\mathrm{M}})_{\mathrm{spiroviolene}}}{(k_{\mathrm{cat}}/K_{\mathrm{M}})_{\mathrm{hedycaryol}}} = \frac{v_{0,\mathrm{spiroviolene}}}{v_{0,\mathrm{hedycaryol}}} \times \frac{[\mathrm{FPP}]}{[\mathrm{GGPP}]} \qquad \textit{Eq.2}$$

Malachite Green Screening of Variants

Freshly purified variants of SvS-A2 were screened for activity using a Malachite Green assay, adapted from Vardakou et al.[26], based on the inorganic pyrophosphate released as a side product of terpene cyclization. As the Malachite Green assay detects inorganic phosphate, inorganic pyrophosphatase was added to the reaction buffer (50 mM Tris-HCl, 5 mM $MgCl_2$, pH 7.4 at 25 °C) to a concentration of 50 mU mL^{-1}. Either FPP or GGPP (50 µM) and a negative control (no substrate) were prepared in 130 µl reaction buffer in a 96 deep-well plate format. The methanol concentration resulting from the addition of 50 µM substrate stock (70% methanol) in all wells corresponds to 1.8% (v/v). The plates were pre-incubated at 30 °C for 10 min without agitation. Subsequently, 20 µl of the different enzyme variants (in a range of ca. 0.2 µM to 3.9 µM) or buffer (enzyme-free negative controls) were added to the reaction wells to reach a final volume of 150 µl. Each reaction was run in triplicate and a standard dilution series ranging from 0.2 µM to 50 µM of dibasic sodium pyrophosphate was added to the plate to ensure that obtained signals are in the linear range of absorbance detection. The plates were sealed with a breathable film and shaken vigorously for a duration of 2 hat 30 °C. Malachite Green dye stock solution was used to freshly prepare Malachite Green development solution as described[26] and filtered through a 0.22 µm syringe filter before use. A colorimetric development plate was prepared by adding 100 µl of reaction buffer without pyrophosphatase into a fresh 96-well clear microplate (Greiner, Austria) and adding 36 µl of Malachite Green development solution to each well. Fifty microliter solution from each well on the reaction plate were transferred to the prepared development plate using a multi-channel pipette. After ca. 1 min, 7.5 µl of 34% (w/v) sodium citrate solution was added to each well as a color stabilizer. The development reaction was incubated for 20 min in the dark at room temperature on a plate shaker (750 rpm) before the absorbance was measured at 623 nm on a Spark plate reader (Tecan, Switzerland). The obtained signals were blanked against controls containing no enzyme and no substrate and signals were normalized to the enzyme concentration employed in each well. The sensitivity threshold was determined as 0.05 absorbance units after blanking and signals obtained below this threshold were considered as noise (represented with a dash in Figure 5).

a

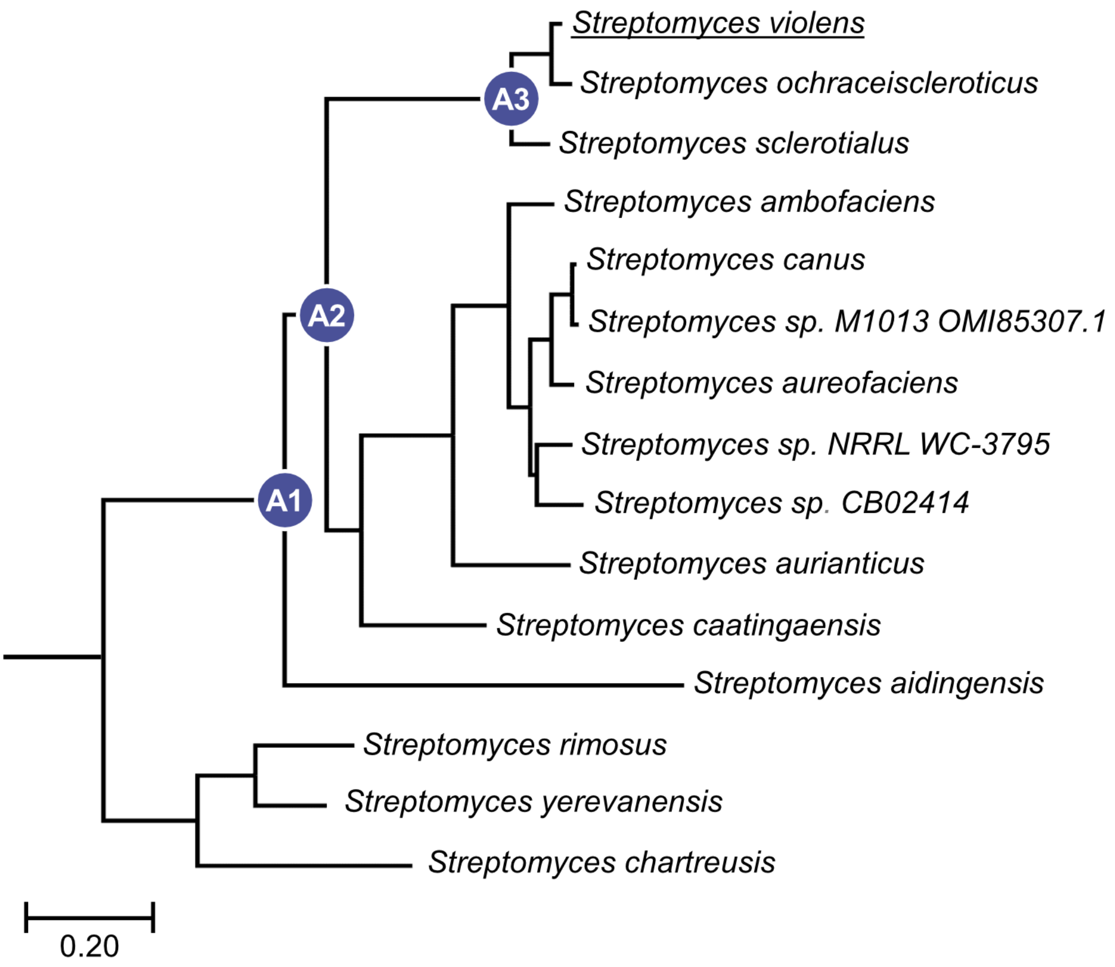

b

MAMTVNEIDLPPIFCPLESARHPRAHLVDERAREWIRTSPMCTTDEERTWVAASCSTDFFARFAPDAATDDRLLWT
SLWVYWGFAFDDHRCDNGPFSNRPAAFSALAGRVQRALEAPSARDESDGFIPALQEIAAQFRSFGTPLQVRRFAAA
HRAWLSGVTWQIGNAAAGRMPGLDEYVAMRLLSAGGEPPFAMLELATGLEVPAQDLERPAVRALTEMAIMVAAL
DNDRHSLRKELARGQTDQNVYSVLMQETGLPLQEAVAAATRLRDRVLLRFMAVHDRVRPGAGLELSTYLQGLRYGI
RGNAEWGLRVPRYLSLGRVPDPMDEAPLEWAESPADDDRSAPRGLPTVAWWWDDALLGV

c

MAMTVTEVDLPPIYCPLESAIHPRVHEVEKRAVEWIRRSGMCASEEERAWVIATHSADFFARFAPTAADEDRLLAT
SLWVYWGFAFDDARCDNGPLSTRPAQFNALAGRVQRALEAPSAEDNGDRFVPALQDIARRFRSFGTPTQVRRFV
HAHRAWLSGVAWQIGNQARGHMPGLDDYLAMRLLSAGGEPTFAMLEIATGAEVPDREMHRPAVRALTEMAIM
VAALDNDRHSLRKELSRGHTDQNIYSVLMHHRGMSLQEAVEEATKLRDRILLRFLELHDRVRPGAGAELSTYLQGL
RHGIRGNAEWGLRVPRYLSLGRVPDPMEDAPLTWAESPSDSSPSPLPGAPSIAWWWDDALLGA

Figure S1. Ancestral sequence reconstruction of SvS. (a) Phylogenetic tree of streptomycetal spiroviolene synthase homologs, underlying the reconstruction of SvS-A2. The relation of SvS-A2 to other putative ancestors (SvS-A1 and SvS-A3) and extant enzymes is shown. Adapted from Hendrikse, N.M et al.[1]: Ancestral diterpene cyclases show increased thermostability and substrate acceptance. The FEBS Journal. 2018. 285. 4660-4672. Copyright John Wiley & Sons, Inc. Reproduced with permission. **(b)** Amino acid sequence of SvS-WT used as a query for the BLAST search.[1] **(c)** Reconstructed amino acid sequence of SvS-A2.[1] All proteins were expressed with an N-terminal His_6-tag.

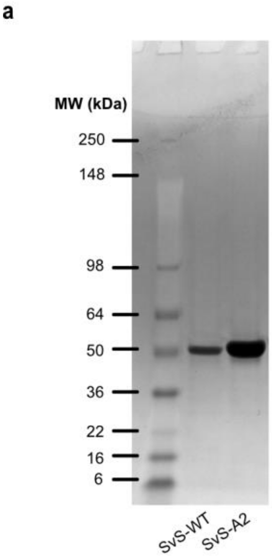

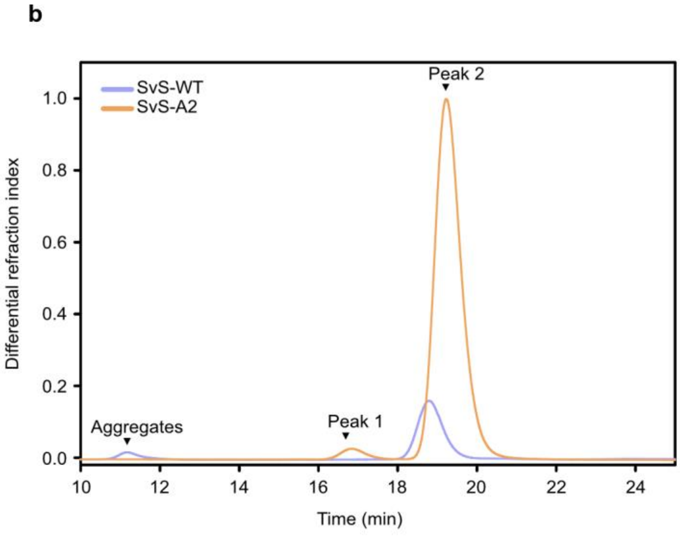

	mg L^{-1} culture
SvS-WT	6.6
SvS-A2	45

	Peak1		Peak2	
	kDa	%	kDa	%
SvS-WT	-	-	83	100
SvS-A2	187	2	79	98

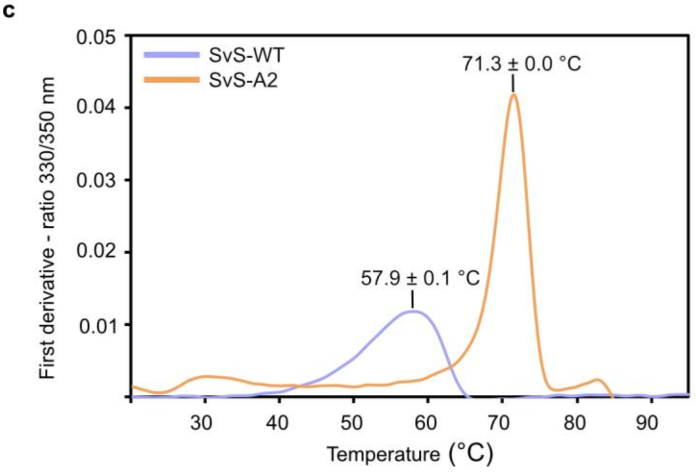

Figure S2. **Stability and solubility of extant and reconstructed ancestral SvS. (a)** SDS-PAGE of His-tag purified, desalted and concentrated extant and reconstructed ancestral spiroviolene synthase (molecular weight ca. 41 kDa), 1:20 dilution from concentrates. Yield of soluble protein determined for extant and reconstructed ancestral spiroviolene synthase, given as mg L^{-1} bacterial expression culture. **(b)** Molecular weights were determined by analytical size exclusion chromatography using multi angle light scattering. Determined molecular weights of the different peaks as well as respective mass fractions (in % of soluble protein, aggregates excluded) are given in the table. Monomeric weight ca. 41 kDa **(c)** Thermal melting curves of the extant and reconstructed ancestral enzyme as determined by nano-DSF (T_m values indicated). The tryptophan content is comparable in SvS-A2 (3.0%) and SvS-WT (3.3%).

Table S1. Crystallization Conditions, Crystal Treatment and General Structure Attributes.

Protein Variant [a]	Crystallization condition	Cryoprotection / Ligand soaking	Resolu tion	Modeled residue range (A,B chains)	Missing residues / gaps
SvS-A2	0.2M Na-phosphate<br>0.1M Bis-Tris-Propane (BTP) pH 6.5<br>20% PEG 3350	none	2.30 Å	A 9-359<br>B 10-359	A 234-240, 312-328,<br>B 90-97, 234-243, 313-328
SvS-A2 (W79F_G83L)	0.2M Na-phosphate<br>0.1M BTP pH 7.2<br>12% PEG 3350	0.2M Na-phosphate<br>0.1M Bis-Tris-Propane pH 7.2<br>20% PEG 3350<br>5mM $MgCl_2$<br>5 mM FPP	2.27Å	A 9-359<br>B 10-360	A 234-241, 312-326<br>B 89-97, 234-241, 313-326
SvS-A2 (205-209: DREMH/AQDLE)	0.2M Na-phosphate<br>0.1M BTP pH 6.5<br>20% PEG 3350	none	2.38 Å	A 9-359<br>B 10-359	A 234-241, 314-325<br>B 89-97, 235-242, 314-323
SvS-A2 (A224I)	0.2M Na-phosphate<br>0.1M BTP pH 6.5<br>12% PEG 3350	0.2M Na-phosphate<br>0.1M BTP: Bis-Tris-Propane pH 6.5<br>20% PEG 3350<br>5mM $MgCl_2$<br>5 mM FPP	2.60 Å	A 9-360<br>B 8-360	A 90-93, 236-240, 312-326<br>B 90-96, 235-242, 313-326
SvS-A2 (W156Y)	0.2M Na-phosphate<br>0.1M BTP pH 6.3<br>18% PEG 3350	0.2M Na-phosphate<br>0.1M Bis-Tris-Propane pH 6.3<br>24% PEG 3350<br>5mM $MgCl_2$<br>5 mM FPP	2.40 Å	A 9- 360<br>B 9-361	A 235-240, 314-325<br>B 90-97, 235-242, 314-325

[a] The indicated SvS-A2 variants (W79F, 205-209:DREMH/AQDLE, A224I, W156Y) carry an additional A89H exchange.

Table S2. Modeling Scores Obtained from YASARA upon Homology Modeling of SvS-WT.

Model	Template	Type	Z-score	Comment[a]
SvS-WT-Hom1	SvS-A2 complexed to GGPP	Dihedrals	-0.429	Good
		Packing 1D	-0.538	Good
		Packing 3D	-0.732	Good
		Overall	-0.612	Good
SvS-WT-Hom2	SvS-A2 surface variant complexed to GGPP	Dihedrals	+0.541	Optimal
		Packing 1D	-0.531	Good
		Packing 3D	-0.123	Good
		Overall	-0.186	Good
SvS-WT-Hom3	PDB-ID: 4OKZ	Dihedrals	-0.266[b]	Good
		Packing 1D	-0.803[b]	Good
		Packing 3D	-0.400[b]	Good
		Overall	-0.538[b]	Good

[a] Z-score classification used according to YASARA output files: "Good" denotes a Z-score between -1 and 0, "Optimal" denotes a Z-score greater than 0. [b] 15 terminal residues were modeled but excluded from the Z-score calculation by YASARA.

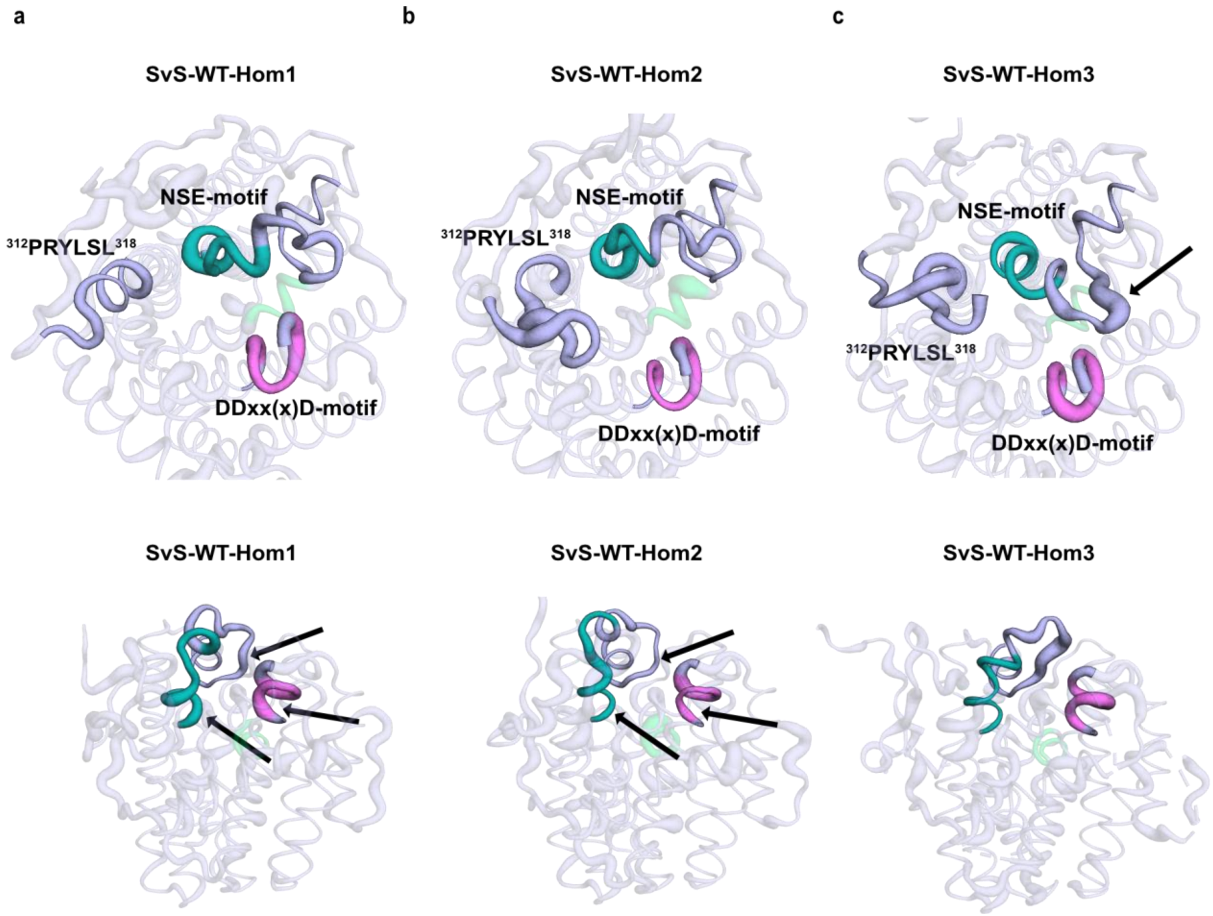

Figure S3. Comparison of the different homology models of SvS-WT. (a) Top and side views of SvS-WT-Hom1 (based on SvS-A2) **(b)** SvS-WT-Hom2 (based on SvS-A2 surface variant) and **(c)** SvS-WT-Hom3 (based on PDB-ID 4OKZ). The coloring of motifs is as in Figure 1. The metal-binding DDxx(x)D motif (helix C) and NSE motif (helix H), as well as the capping loop (helix K) are shown opaque and are labelled. The thickness of the cartoon represents the Z-score at each residue, with thick cartoon indicating a lower Z-score and thin cartoon indicating a higher Z-score. In the top row an arrow in SvS-WT-Hom3 indicates the structural alterations in the metal binding site compared to the other models. In the bottom row the capping loop is shown transparent for clarity. Three arrows in the SvS-WT-Hom1 and SvS-WT-Hom2 models highlight regions of increased local Z-score (i.e. thinner cartoon) in SvS-WT-Hom2 compared to SvS-WT-Hom1.

Table S3. Active Site Conservation Between SvS-WT, SvS-A2, Hedycaryol Synthase and Selinadiene Synthase.

Helix [a]	Position in SvS- WT	Position in SvS-A2	(Putative) function in SvS-A2[b]	Position in HecS[16] [c]	Function in HecS[16]	Position in SdS[5] [d]	Function in SdS[5]
**B1**	W50	W50		R46		K46	
	V51	V51		M47		L47	
**X**	S54	T54		T50		Q50	
**B2**	S56	S56		A52		I52	
	F59	F59	π interaction with **3**,**4**	S55	*S55W inactive at pH 7.5/8*	F55	*F55Y active, F55W active, F55L active (product distribution altered for all)*
	F60	F60		G56		S56	
	F63	F63		V59		I59	
**C**	T76	T76		G71		L71	
	S77	S77		L72		A72	
	W79	W79	π interaction with **2**, *W79F_G83L inactive*	W74		F74	
	V80	V80	*V80I GGPP specific*	I75		I75	
	Y81	Y81		A76		L76	
	W82	W82	π interaction with **2**	L77		W77	
	G83	G83	backbone carbonyl stabilizes **2**, *W79F_G83L inactive*	T78		L78	
*	F84	F84	π interaction with **2**, **13**	F79		F79	*F79L linear products F79Y active, F79W active (product distribution altered for both)*
	F86	F86		I81		V81	
*	D87	D87	Mg^{2+} coordination, Water activation for quenching of **13**	D82	*D82N inactive at pH 7, D82N low activity at pH 8.5*	D82	
*	D88	D88	Mg^{2+} coordination	D83		D83	*D83N almost inactive, D83E altered product distribution*
	C91	C91		unres-olved		C86	
	D92	D92	Mg^{2+} coordination	unres-olved		E87	
	N93	N93		unres-olved		E88	
**F**	W156	W156	*W156Y GGPP specific with higher activity*	F149	*F149L inactive at pH 7, F149L low activity at pH 8.5, F149W highly active at pH 7 and 8.5*	Y152	*Y152F active, Y152L active, Y152W active, (product distribution altered for all)*
	V160	V160		T152		V156	
	Q163	Q163		E156		E159	*E159D active, E159Q active (product distribution altered for both)*
**G1** *	R182	R182	"PPi sensor"[5]	R175	"PPi sensor"	R178	"PPi sensor" *R178Q inactive, R178K altered product distribution*

	S185	S185		S178		D181	"Linker residue" *D181S active, D181N active*
**G-kink**	A186	A186	"Effector residue"[5], backbone carbonyl stabilizes **4, 6**, **10**, backbone carbonyl stabilizes **12** for anti-Markovnikov addition	V179	"Effector residue"	G182	"Effector residue" *G182A active, G182V inactive, G182P inactive*
	G187	G187	*G187A GGPP specific*	G180		A183	*A183V inactive, A183F inactive*
	G188	G188	*G188L FPP specific, G188T inactive, G188V FPP specific*	M181	*M181K inactive at pH 7/8.5, M181H inactive at pH 7, M181H low activity at pH 8.5*	T184	
**G2**	P190	P190	*P190V GGPP specific*	W183		V186	
**X**	P191	T191	*T191V GGPP specific, T191P GGPP specific with higher activity*	L184		V187	
	F192	F192		W185		L188	
	M194	M194		L187		M190	
**X**	L197	I197	*I197L GGPP specific*	L190		M193	
**H**	T217	T217		R210		A213	
	A220	A220		G213		A216	
	I221	I221		A214		S217	
	A224	A224	*A224I FPP specific with higher activity*	I217		I220	
	A225	A225	*A225F inactive, A225L inactive, A225T inactive, A225V GGPP specific*	A218		T221	
*	N228	N228	Mg^{2+} coordination	N221		N224	
*	D229	D229		D222		D225	
	H231	H231		F224		F227	
*	S232	S232	Mg^{2+} coordination	S225		S228	
	E236	E236		unresol ved		E232	
**K**	G297	G297		N298		S293	
	G301	G301	*G301F inactive*	N302		F297	
	I302	I302		L303		I298	
	N305	N305		Q306		A301	
*	W308	W308	π interaction with **3**	W309	*W309F active, W309L active, W309Y active all at pH 7.5/8*	W304	*W304F active, W304Y active, W304L altered product distribution*
	G309	G309		H310	*H310S inactive at pH 7/8.5*	G305	

[a] Helix numbering according to SvS-A2. Positions that differ between SvS-WT and SvS-A2 are indicated with X, residues that are conserved across all four enzymes are marked with asterisk. [b] Intermediates stabilized by putative π interactions, suggested from modelled pathways (Figure 4, Figure S7) are indicated in brackets; summary from enzyme library experiments indicated in italics. [c] PDB-ID: 4MC3[16]; structural homolog to SvS-A2 generating the same product from FPP (35.5% sequence similarity). [d] PDB-ID: 4OKZ[5]; closest sequence homolog of known structure to SvS-A2 (45.2% sequence similarity).

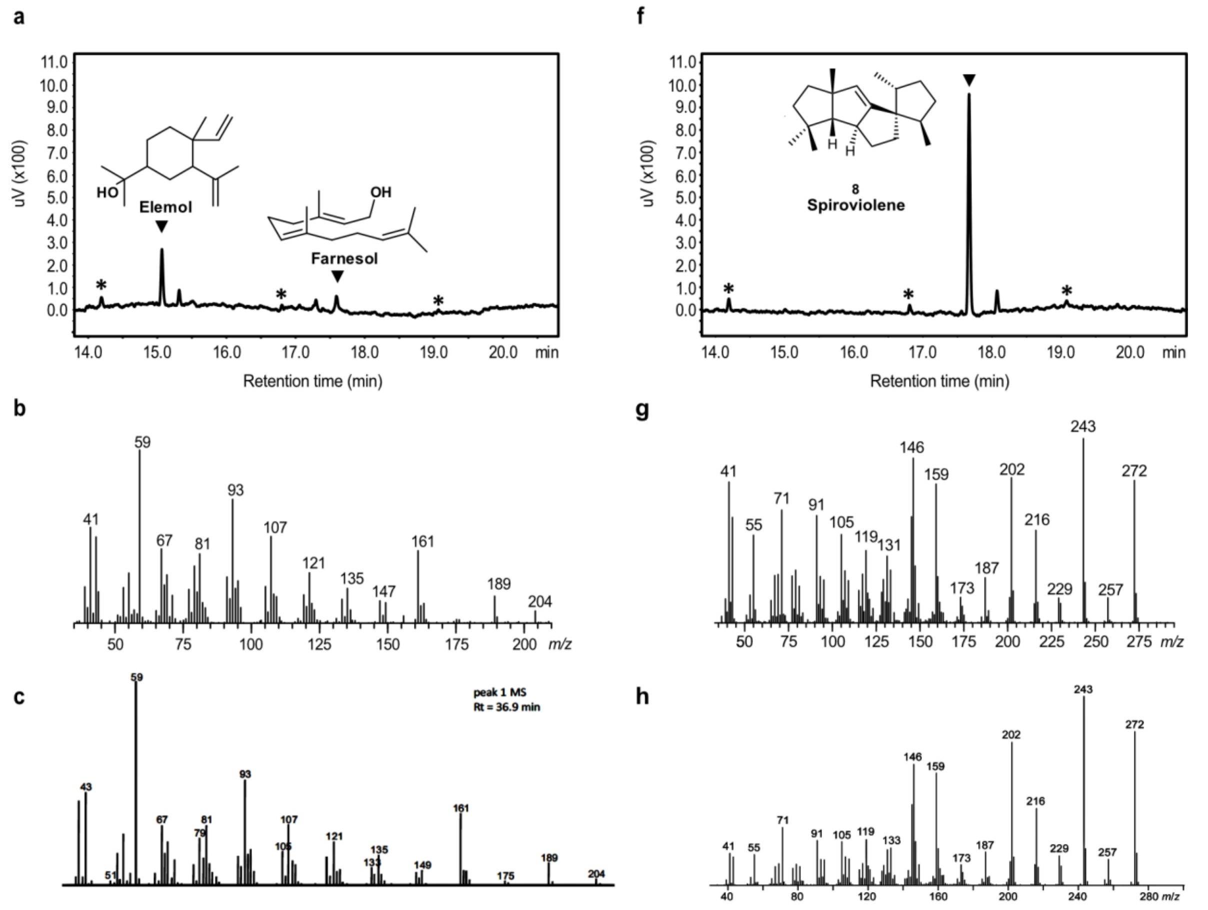

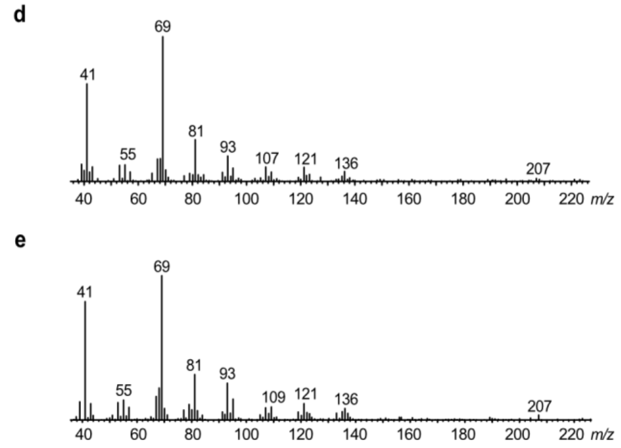

i

Compound	LRI[a]	Reference LRI[b]
elemol [hedycaryol[c]]	1560	1547[27] [1550[16]]
(*2E,6E*)-farnesol	1727	1726[d]
spiroviolene	1736	1724[15]

[a] Linear retention indices are measured on a Rxi-5ms column with FID-detection as described in the Methods section. [b] Literature indices are from DB-5 or HP-5MS columns, which are equivalent to Rxi-5ms column. [c]Hedycaryol is detected as its thermal rearrangement product elemol, generated in situ in the GC-injector[24]. [d] The given LRI is obtained from an experimental standard run on the same column. Differences may occur due to rounding.

Figure S4. Characterization of SvS-A2 products. Product peaks are marked by arrows and peaks appearing in the solvent blank are marked by asterisks. Controls with either protein or substrates excluded did not show product peaks (not shown). **(a)** GC-FID chromatogram of SvS-A2 reaction with 60 µM FPP. **(b)** Mass spectrum of major sesquiterpene product from SvS-A2 identified at 15.1 min elution time. **(c)** Mass spectrum of elemol adapted from Hattan, J. et al.[28]: Identification of a novel hedycaryol synthase gene isolated from Camellia brevistyla flowers and floral scent of Camellia cultivars. Planta. 2016. 243. 959-972. Copyright Springer Science+Business Media. Reproduced with permission. **(d)** Mass spectrum of sesquiterpene side product from SvS-A2 identified at 17.6 min elution time. **(e)** Mass spectrum of (*2E,6E*)-farnesol reference standard. **(f)** GC-FID chromatogram of SvS-A2 reaction with 60 µM GGPP using the same enzyme preparation as in (a). **(g)** Mass spectrum of major diterpene product from SvS-A2 identified at 17.7 min elution time. **(h)** Mass spectrum of spiroviolene adapted from Rabe, P. et al.[15]: Mechanistic Investigations of Two Bacterial Diterpene Cyclases: Spiroviolene Synthase and Tsukubadiene Synthase. Angew Chem Int Ed Engl. 2017. 56. 2776-2779. Copyright 2017 Wiley-VCH Verlag GmbH & Co. KGaA. Reproduced with permission. **(i)** Linear retention indices and literature values.

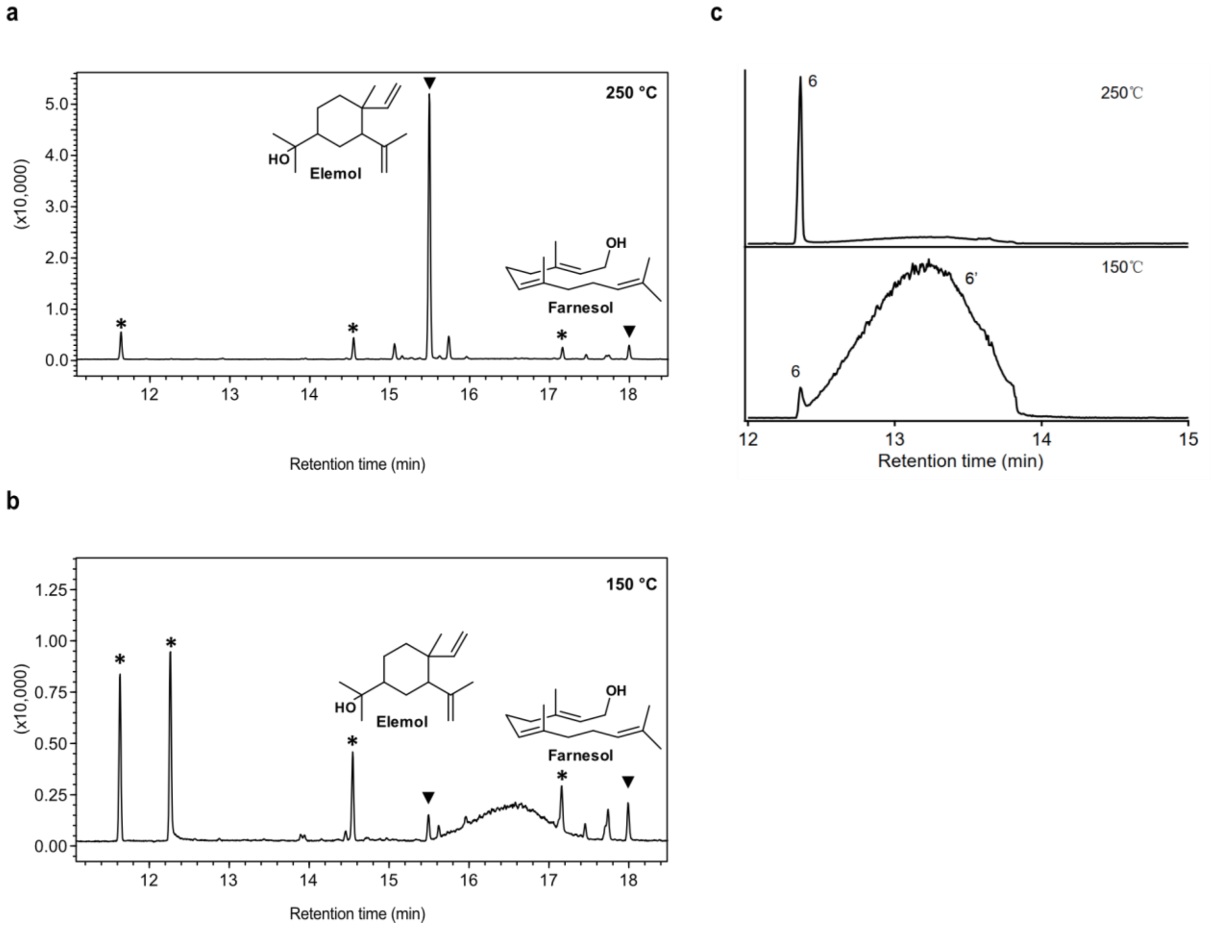

Figure S5. **Thermal analysis of elemol formation. (a)** GC-MS selected ion count chromatogram (*m*/*z* values 59, 93, 161) of SvS-A2 reaction with 60 µM FPP using the GC method described in the Methods section (injection at 250 °C). Identity of elemol (thermal rearrangement product from **14**) and farnesol (hydroxylation product from from **10**) was verified by complementary full mass range scans (Figure S4). Product peaks are marked by arrows and peaks appearing in the solvent blank are marked by asterisks. **(b)** Zoomed selected ion chromatogram (*m*/*z* values 59, 93, 161) of identical sample injected at 150 °C. Product peaks are marked by arrows and peaks appearing in the solvent blank are marked by asterisks. **(c)** GC chromatogram showing thermal rearrangement of hedycaryol to elemol, adapted from Liang, J. et al.[29]: Direct production of dihydroxylated sesquiterpenoids by a maize terpene synthase. Plant J. 2018. 94. 847-856. Copyright Wiley-Blackwell. Reproduced with permission.

Table S4. **Calculations of Binding Energies Between Monomeric Holoenzymes and Either GGPP or FPP.**

Enzyme	Substrate	ΔG_{bind} (kcal mol^{-1})	$\Delta_{GGPP-FPP}\Delta G_{bind}$ (kcal mol^{-1})
SvS-A2	GGPP	-37.93	-10.69
	FPP	-27.24	
SvS-WT-Hom2	GGPP	-32.39	-8.72
	FPP	-23.67	

Table S5. Key Atom Distances in Intermediates along the Reaction Pathway in the Formation of Spiroviolene.

Reaction intermediate[a]		Enzyme	Reaction	Distance Å	Reaction	Distance Å
1	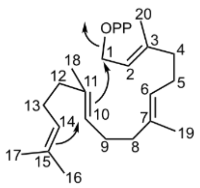	SvS-A2	C10-C14 Cyclization	3.06	C1-C11 Cyclization	4.36
		SvS-WT		3.04		3.91
2	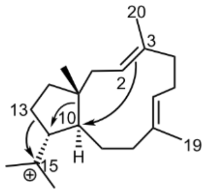	SvS-A2	C2-C10 Cyclization	2.95		
		SvS-WT		2.95		
3	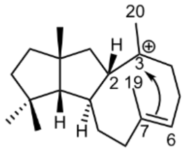	SvS-A2	C3-C6 Cyclization	2.58		
		SvS-WT		2.59		
4	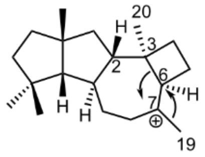	SvS-A2	C3-C7 Ring expansion	2.59	C6-C19 Methyl shift	2.64
		SvS-WT		2.58		2.65
5	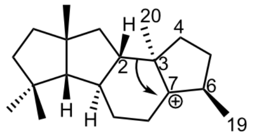	SvS-A2	C2-C7 Ring contraction	2.61		
		SvS-WT		2.53		
6	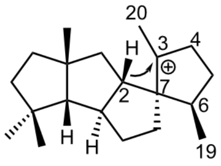	SvS-A2	H2-C3 1,3-hydride migration	2.66		
		Svs-WT		2.63		
7	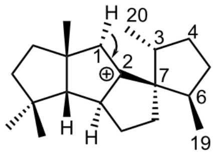	SvS-A2	H1-O_{wat} *pro-R* proton abstraction	3.15	H1-O_{wat} *pro-S* proton abstraction	3.08
		SvS-WT		2.50		2.53

[a] The reaction intermediates complexed to the active site are shown in Figure 4. Numbering of atoms in intermediates is based on the numbering in GGPP.

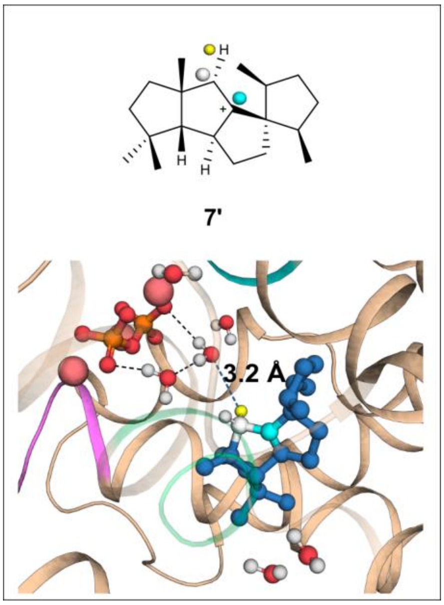

Figure S6. Alternative penultimate intermediate in the GGPP cyclization cascade. Active site from Figure 4, panel **7** superposed on an intermediate derived from a recently reported alternative reaction mechanism for spiroviolene formation.[30-31] The intermediate with the methyl-groups at C3 and C19 (numbering based on numbering in GGPP) in *syn* configuration fits in the active site of both SvS-WT and SvS-A2.

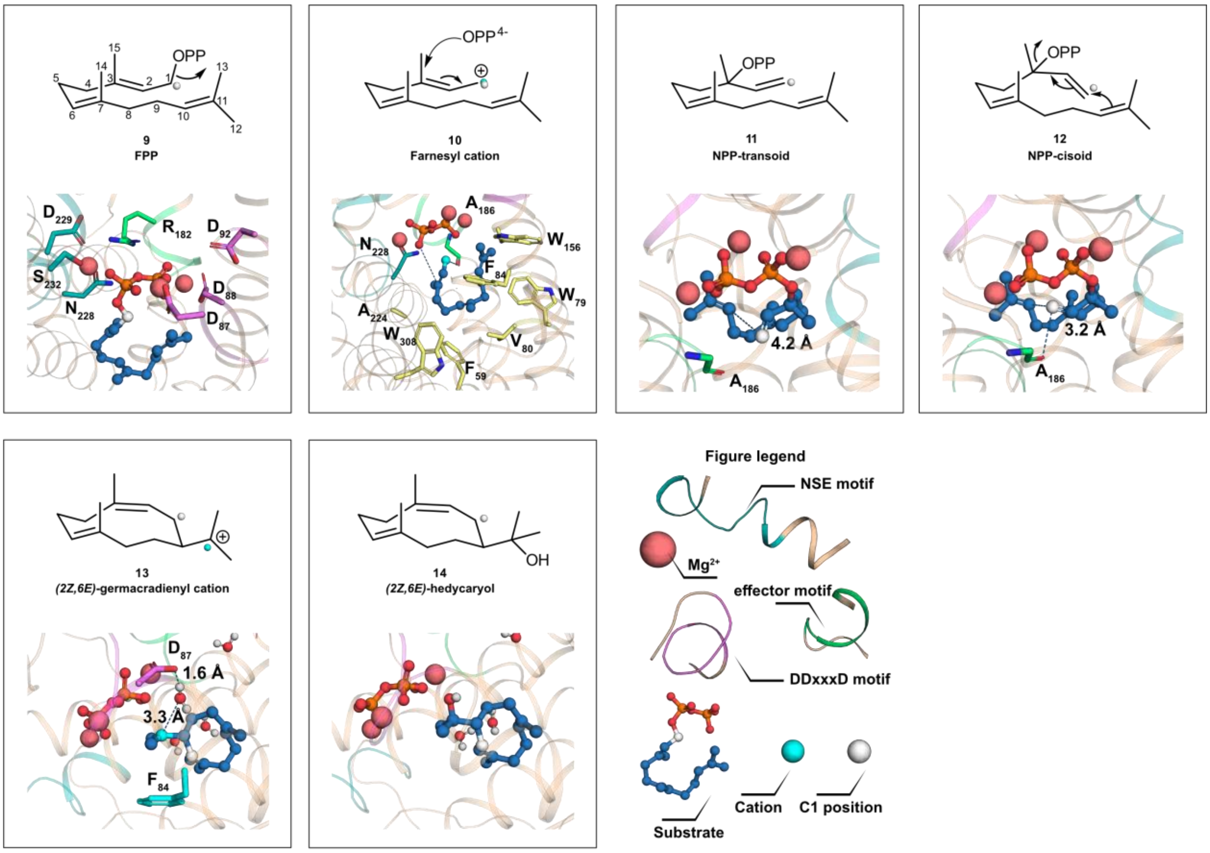

Figure S7. Snapshots of the SvS-A2 catalyzed cyclization of FPP to hedycaryol. Key distances for bond forming/breaking reactions are shown as blue dotted lines, respective distances for SvS-A2 and SvS-WT are given in Table S6. The proposed electron flow characterized from another hedycaryol synthase is represented with conventional arrows in the 2D depiction.[16] Residues involved in complexing metals and pyrophosphate in **9** are shown as sticks and labelled, but omitted for clarity in other panels. The hydrophobic cage around the intermediate is shown as sticks in **10** and for clarity only residues in close proximity of the cation are shown as sticks in the following panels. Anti-Markovnikov addition of the double bond at C1 in **12** is assisted by stabilization of the forming charge by the backbone carbonyl of Ala186 (shown as green sticks and by dashed line). Individual residues involved in cationic π-interactions are highlighted as cyan sticks. For nucleophilic water attack on **13** water molecules within a radius of 9Å of the ligand are shown. One water molecule bridges C11 and Asp87 (distance Owat···C11 = 3.3 Å, distance Hwat···OAsp87 = 1.6 Å). In SvS-WT, the water molecule is located closer to the pyrophosphate moiety than in SvS-A2 (Table S6).

Table S6. Key Atom Distances in Intermediates along the Reaction Pathway in the Formation of Hedycaryol.

Reaction intermediate[a]		Enzyme	Reaction	Distance Å	Other	Distance Å
10	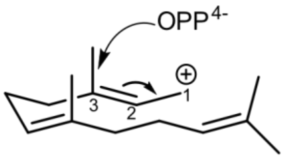	SvS-A2	O_{PPi}-C3 Nucleophilic attack	4.34	Stabilization by C1-CO_{S185}/ C1-CO_{A186}	5.56/ 3.43
		SvS-WT		4.25		2.86/ 6.10
11	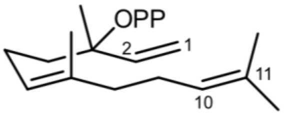	SvS-A2	C1-C10	4.20		
		SvS-WT		4.58		
12	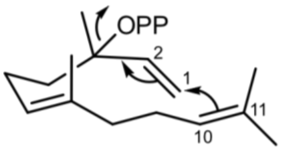	SvS-A2	C1-C10 Cyclization	3.15	Stabilization by C1-CO_{S185}/ C1-CO_{A186}	4.46/ 3.33
		SvS-WT		4.15		3.34/ 5.34
13	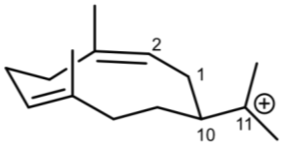	SvS-A2	$C11^{+}$-O_{wat} Nucleophilic attack	3.33	H_{wat}-O_{D87}/ H_{wat}-O_{PPi}	1.60/ 3.70
		SvS-WT		2.94		1.59/ 1.50

[a]The reaction intermediates complexed to the active site are shown in Figure S7. Numbering of atoms in intermediates is based on the numbering in FPP.

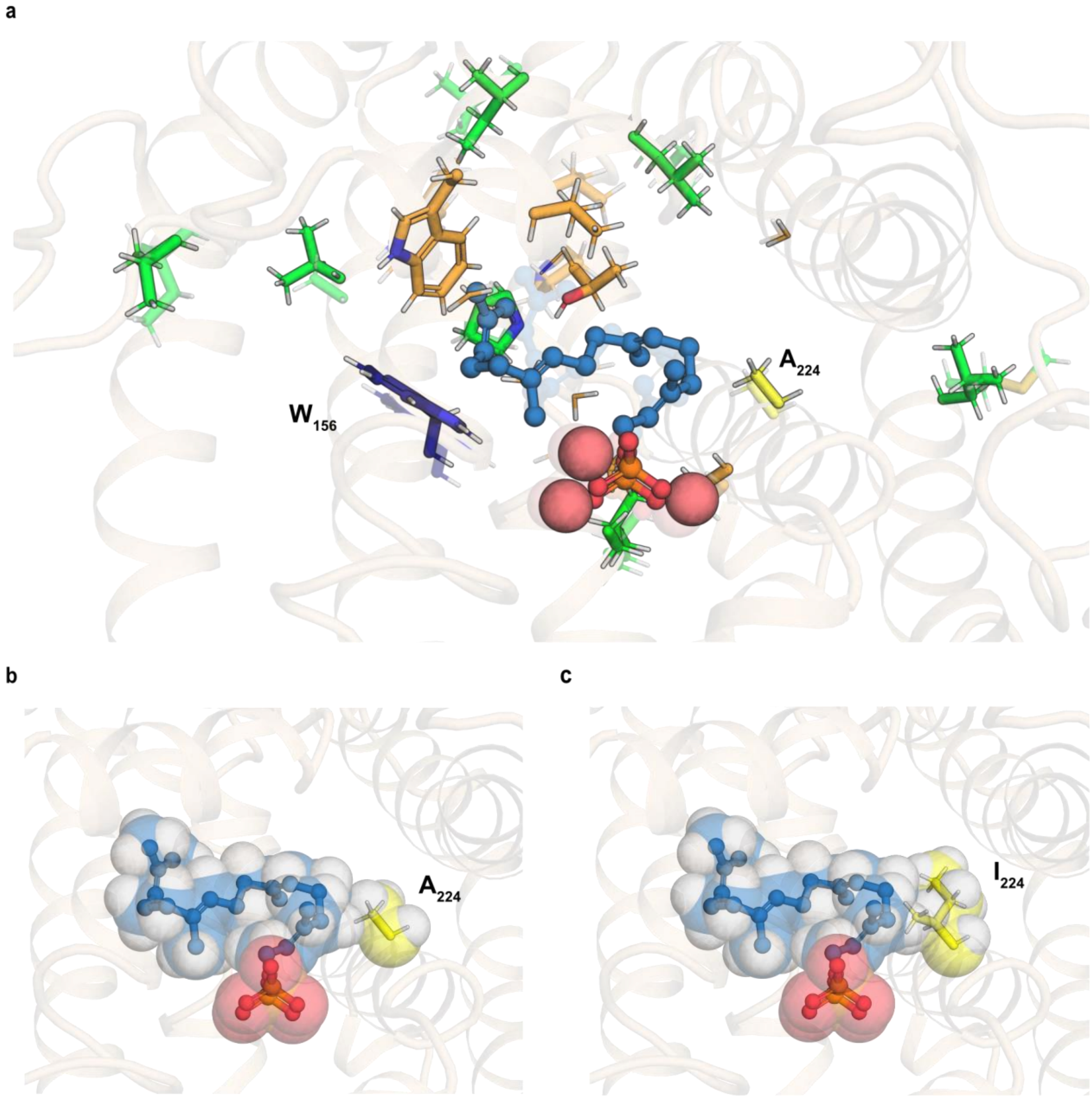

Figure S8. Overview of SvS-A2 variant library. (a) Positions targeted for redesign in the library are shown in sticks in the GGPP-docked model of SvS-A2. Respective positions in the substrate docked homology model of SvS-WT are superposed (shown as transparent sticks). The docked substrate is shown as blue sticks and spheres and the tri-metal ion cluster is shown as enlarged pink spheres. Positions lining the active site are colored orange and targeted secondary shell positions are colored green. The key specificity-switch residues identified in the library are labeled and colored in dark blue (Trp156Tyr to achieve GGPP specificity) and yellow (Ala224Ile to achieve FPP specificity). **(b)** The SvS-A2 active site is shown with GGPP docked (shown in dark blue sticks and transparent spheres). The side chain of Ala224 is shown as yellow sticks and transparent spheres. **(c)** The crystal structure of SvS-A2 Ala224Ile is superposed onto (b) and the side chain of Ile224 is shown as yellow sticks and transparent spheres, highlighting the steric clash that likely prevents cyclization of GGPP in the active site of the Ala224Ile variant.

Table S7. Brief Description of SvS-A2 Variant Library.

Position in SvS-A2	SvS-A2 variants[a]	Position in SvS-WT
	Affecting sterics of the active site	
W79, G83	W79F_G83L	W79, G83
V80	V80I	V80
W156	W156Y	W156
A186	A186G	A186
G187	G187A	G187
G188	G188L, G188T, G188V	G188
T191	T191P, T191V	P191
A224	A224I	A224
A225	A225F, A225L, A225T, A225V	A225
G301	G301F	G301
	Secondary shell mutations	
V127	V127I	I127
V150	V150A	A150
L183	L183F, L183W	L183
P190	P190V	P190
L195	L195F	L195
I197	I197L	L197
L277	L277M	M277

[a] All variants carry an additional A89H exchange to preserve the metal binding site as in SvS-WT.

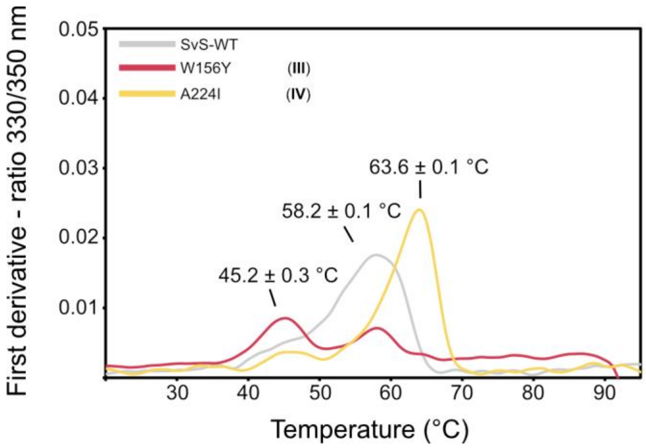

Figure S9. Thermal stability of specificity-switch variants of SvS-WT. Melting temperatures were determined by nano-DSF as the maximum of the derivative of the 330/350 nm ratio (technical replicates, one representative trace is shown, T_m values indicated).

Supporting References

1. Hendrikse, N. M.; Charpentier, G.; Nordling, E.; Syrén, P.-O., Ancestral diterpene cyclases show increased thermostability and substrate acceptance. *FEBS J.* **2018,** *285* (24), 4660-4673.
2. Kabsch, W., XDS. *Acta Crystallogr., Sect. D: Biol. Crystallogr.* **2010,** *66* (Pt 2), 125-32.
3. Winn, M. D.; Ballard, C. C.; Cowtan, K. D.; Dodson, E. J.; Emsley, P.; Evans, P. R.; Keegan, R. M.; Krissinel, E. B.; Leslie, A. G.; McCoy, A.; McNicholas, S. J.; Murshudov, G. N.; Pannu, N. S.; Potterton, E. A.; Powell, H. R.; Read, R. J.; Vagin, A.; Wilson, K. S., Overview of the CCP4 suite and current developments. *Acta Crystallogr., Sect. D: Biol. Crystallogr.* **2011,** *67* (Pt 4), 235-42.
4. Vagin, A.; Teplyakov, A., Molecular replacement with MOLREP. *Acta Crystallogr., Sect. D: Biol. Crystallogr.* **2010,** *66* (Pt 1), 22-5.
5. Baer, P.; Rabe, P.; Fischer, K.; Citron, C. A.; Klapschinski, T. A.; Groll, M.; Dickschat, J. S., Induced-fit mechanism in class I terpene cyclases. *Angew. Chem., Int. Ed.* **2014,** *53* (29), 7652-6.
6. Perrakis, A.; Morris, R.; Lamzin, V. S., Automated protein model building combined with iterative structure refinement. *Nat. Struct. Biol.* **1999,** *6* (5), 458-63.
7. Emsley, P.; Lohkamp, B.; Scott, W. G.; Cowtan, K., Features and development of Coot. *Acta Crystallogr., Sect. D: Biol. Crystallogr.* **2010,** *66* (Pt 4), 486-501.
8. Murshudov, G. N.; Skubák, P.; Lebedev, A. A.; Pannu, N. S.; Steiner, R. A.; Nicholls, R. A.; Winn, M. D.; Long, F.; Vagin, A. A., REFMAC5 for the refinement of macromolecular crystal structures. *Acta Crystallogr., Sect. D: Biol. Crystallogr.* **2011,** *67* (Pt 4), 355-67.
9. Krissinel, E.; Henrick, K., Inference of macromolecular assemblies from crystalline state. *J. Mol. Biol.* **2007,** *372* (3), 774-97.
10. Williams, C. J.; Headd, J. J.; Moriarty, N. W.; Prisant, M. G.; Videau, L. L.; Deis, L. N.; Verma, V.; Keedy, D. A.; Hintze, B. J.; Chen, V. B.; Jain, S.; Lewis, S. M.; Arendall, W. B., 3rd; Snoeyink, J.; Adams, P. D.; Lovell, S. C.; Richardson, J. S.; Richardson, D. C., MolProbity: More and better reference data for improved all-atom structure validation. *Protein Sci.* **2018,** *27* (1), 293-315.
11. Krieger, E.; Joo, K.; Lee, J.; Lee, J.; Raman, S.; Thompson, J.; Tyka, M.; Baker, D.; Karplus, K., Improving Physical Realism, Stereochemistry, and Side-Chain Accuracy in Homology Modeling: Four Approaches that Performed Well in CASP8. *Proteins* **2009,** *77*, 114-122.
12. Krieger, E.; Vriend, G., YASARA View—molecular graphics for all devices—from smartphones to workstations. *Bioinformatics* **2014,** *30* (20), 2981-2982.
13. *Molecular Operating Environment (MOE)*, version 2015.10; Montreal, QC, 2015.
14. Case, D. A.; Darden, T. A.; Cheatham, T. E.; Simmerling, C. L., III; Wang, J.; Duke, R. E.; Luo, R.; Crowley, M.; Walker, R. C.; Zhang, W.; Merz, K. F.; Paesani, F.; Vanicek, J.; Wu, X. M.; Brozell, S. R.; Steinbhrecher, H.; Gohlke, H.; Yang, L. X.; Tan, C.; Mongan, J.; Hornak, V.; Cui, G.; Mathews, D. H.; Seetin, M. G.; Sagui, C.; Babin, V.; Kollman, P. A. *AMBER10*, version 2015.10; San Francisco, 2008.
15. Rabe, P.; Rinkel, J.; Dolja, E.; Schmitz, T.; Nubbemeyer, B.; Luu, T. H.; Dickschat, J. S., Mechanistic Investigations of Two Bacterial Diterpene Cyclases: Spiroviolene Synthase and Tsukubadiene Synthase. *Angew. Chem., Int. Ed.* **2017,** *56* (10), 2776-2779.
16. Baer, P.; Rabe, P.; Citron, C. A.; de Oliveira Mann, C. C.; Kaufmann, N.; Groll, M.; Dickschat, J. S., Hedycaryol synthase in complex with nerolidol reveals terpene cyclase mechanism. *ChemBioChem* **2014,** *15* (2), 213-6.
17. Bowie, J. U.; Luthy, R.; Eisenberg, D., A method to identify protein sequences that fold into a known three-dimensional structure. *Science* **1991,** *253* (5016), 164-70.
18. Luthy, R.; Bowie, J. U.; Eisenberg, D., Assessment of protein models with three-dimensional profiles. *Nature* **1992,** *356* (6364), 83-5.
19. Bujnicki, J.; Rychlewski, L.; Fischer, D., Fold-recognition Detects an Error in the Protein Data Bank. *Bioinformatics* **2002,** *18*, 1391-1395.
20. Colovos, C.; Yeates, T. O., Verification of protein structures: patterns of nonbonded atomic interactions. *Protein Sci.* **1993,** *2* (9), 1511-9.

21. Ramachandran, G. N.; Sasisekharan, V., Conformation of polypeptides and proteins. *Adv. Protein Chem.* **1968,** *23*, 283-438.
22. Lovell, S. C.; Davis, I. W.; Arendall, W. B., 3rd; de Bakker, P. I.; Word, J. M.; Prisant, M. G.; Richardson, J. S.; Richardson, D. C., Structure validation by Calpha geometry: phi,psi and Cbeta deviation. *Proteins* **2003,** *50* (3), 437-50.
23. Vandendool, H.; Kratz, P. D., A Generalization of the Retention Index System Including Linear Temperature Programmed Gas-Liquid Partition Chromatography. *J. Chromatogr.* **1963,** *11*, 463-71.
24. Jones, R. V. H.; Sutherland, M. D., Hedycaryol, the precursor of elemol. *Chem. Commun.* **1968,** (20), 1229-1230.
25. de Saint Laumer, J. Y.; Leocata, S.; Tissot, E.; Baroux, L.; Kampf, D. M.; Merle, P.; Boschung, A.; Seyfried, M.; Chaintreau, A., Prediction of response factors for gas chromatography with flame ionization detection: Algorithm improvement, extension to silylated compounds, and application to the quantification of metabolites. *J. Sep. Sci.* **2015,** *38* (18), 3209-3217.
26. Vardakou, M.; Salmon, M.; Faraldos, J. A.; O'Maille, P. E., Comparative analysis and validation of the malachite green assay for the high throughput biochemical characterization of terpene synthases. *MethodsX* **2014,** *1*, 187-96.
27. Choi, H. S., Character impact odorants of Citrus Hallabong [(C. unshiu Marcov x C. sinensis Osbeck) x C. reticulata Blanco] cold-pressed peel oil. *J Agric Food Chem* **2003,** *51* (9), 2687-92.
28. Hattan, J.; Shindo, K.; Ito, T.; Shibuya, Y.; Watanabe, A.; Tagaki, C.; Ohno, F.; Sasaki, T.; Ishii, J.; Kondo, A.; Misawa, N., Identification of a novel hedycaryol synthase gene isolated from Camellia brevistyla flowers and floral scent of Camellia cultivars. *Planta* **2016,** *243* (4), 959-72.
29. Liang, J.; Liu, J.; Brown, R.; Jia, M.; Zhou, K.; Peters, R. J.; Wang, Q., Direct production of dihydroxylated sesquiterpenoids by a maize terpene synthase. *Plant J.* **2018,** *94* (5), 847-856.
30. Chi, H. M.; Cole, C. J. F.; Hu, P.; Taylor, C. A.; Snyder, S. A., Total syntheses of spiroviolene and spirograterpene A: a structural reassignment with biosynthetic implications. *Chem. Sci.* **2020,** *11* (40), 10939-10944.
31. Xu, H.; Dickschat, J. S., Revision of the Cyclisation Mechanism for the Diterpene Spiroviolene and Investigations of Its Mass Spectrometric Fragmentation. *ChemBioChem* **2020.** **doi.org/10.1002/cbic.202000682**.
